# Supplementary material for: Forecasting influenza activity using machine-learned mobility map
Source: Nat Commun. 2021 Feb 9;12:726. doi: 10.1038/s41467-021-21018-5 (PMC7873234; doi:10.1038/s41467-021-21018-5)
Supplement: Supplementary file 1 — Supplementary Information [file 41467_2021_21018_MOESM1_ESM.pdf]

Forecasting influenza activity using machine-learned mobility map: Supplementary Material

Srinivasan Venkatramanan[1] Adam Sadilek[2*] Arindam Fadikar[3]
Christopher L. Barrett[1,4] Matthew Biggerstaff[5] Jiangzhuo Chen[1]
Xerxes Dotiwalla[2] Paul Eastham[2] Bryant Gipson[2] Dave Higdon[6]
Onur Kucuktunc[2] Allison Lieber[2] Bryan L Lewis[1] Zane Reynolds[7]
Anil K Vullikanti[1,4] Lijing Wang[1,4] Madhav Marathe[1,4]

[1]Biocomplexity Institute & Initiative, University of Virginia, Charlottesville, VA, USA
[2]Google Inc., Mountain View, CA, USA
[3]Argonne National Laboratory, Lemont, IL, USA
[4]Department of Computer Science, University of Virginia, Charlottesville, VA, USA
[5]Influenza Division, Centers for Disease Control and Prevention, Atlanta, GA, USA
[6]Department of Statistics, Virginia Tech, Blacksburg, VA, USA
[7]Torc Robotics, Blacksburg, VA, USA
*Corresponding author: adsa@google.com

Supplementary Notes 1

There has been extensive work on modeling the spread of infectious diseases in human populations using data on inter-regional mobility. In this section, we highlight a few key articles that represent this body of literature, as they fall into one or more of the following broad categories: (a) use of data on human mobility to model infectious diseases, (b) use of metapopulation models to integrate mobility information, and (c) forecasting seasonal influenza activity at national and regional levels. Although we present these in three sub sections for clarity, we note that some of the literature span across these categories.

Mobility for disease dynamics

Infectious disease spread in human population is facilitated by social contacts, which are in turn influenced by the movement of individuals. When constructing a model of disease dynamics, even in the absence of high quality mobility data, one may resort to standard models such as gravity, radiation, etc. [1] provides an extensive review of such mobility models, and their wide-ranging applications including in the field of epidemiology. Nowadays, datasets that capture movement of individuals at micro and macro scales are increasingly available, some of them in the public domain. For instance, call data records (CDR) [2] provide anonymized individual traces at the spatial resolution of base stations. [3] and [4] show how such mobile phone call records were used to model the spread of dengue and malaria in Pakistan and Kenya respectively. Recently in [5] the authors compare multinational call records, to identify seasonal patterns of mobility between different regions, and its impact on disease spread. Though promising as a source of individual level mobility, there are obvious limitations concerning privacy, access, coverage, and noise in CDRs, which are highlighted in [6]. In the absence of CDRs, one may construct synthetic trajectories for

Supplementary Table 1: Features comparison across data sources for human mobility

	Surveys	CDRs	Transport services	Models	GPS traces
Access	Public	Restricted	Public/Restricted	N/A	Restricted
Coverage	Low	Low	Medium	Global	Global
Temporal resolution	Low	High	Medium	Low	High
Update frequency	Low	High	High	Low	High
Sample size	Low	High	Medium	N/A	High
Demographic bias	Low	Medium	Medium	Low	Medium

individuals using activity schedules derived from time use surveys and assigning individuals to activity locations, as described in [7].

Alternative sources for regional mobility include data released as part of official census surveys, as well as public databases. Unlike mobile phone call records, these sources provide information on population mobility in aggregate, which may have its own pros and cons in the context of epidemic modeling. For instance, [8] compares different mobility proxies for modeling epidemics, including community networks obtained from census, radiation models and mobile phone call records. In [9], the authors use airline networks to capture long-distance mobility and the impact of potential travel restrictions on spread of the 2009 A(H1N1) epidemic. During the same epidemic, authors in [10] used multi-scale mobility models based on a proxy network of dispersing US dollar bills to produce spatially explicit real-time forecasts for the outbreak. Data on public transit networks may also aid in understanding human mobility within a city, as authors in [11] demonstrate using the subway network to model and simulate an influenza epidemic in New York City.

Supplementary Table 1 lists and contrasts the features of different mobility sources.

Metapopulation models for disease dynamics

When attempting to reconstruct the spatial and temporal evolution of epidemics, metapopulation models provide an elegant framework to integrate the population and mobility data to simulate the disease spread. They provide a balance between complex agent-based models which are computationally expensive, and the simple compartmental models which do not capture the spatial heterogeneity in disease dynamics. For instance [12] incorporated the commuting networks of 29 countries and the international airline network within a metapopulation model, and studied the combined effect on spatio-temporal patterns in a global epidemic. In [13], the authors used a similar framework to compare and contrast different large-scale computational approaches to epidemic modeling and showed that the models are in good agreement, thus hinting at the possibility of building hybrid approaches. Finally in [14], the authors theoretically analyzed such metapopulation models and showed that scaling properties of human interactions can affect the dynamics caused by human contacts such as infectious disease dynamics.

Influenza forecasting

Forecasting seasonal influenza, especially within the United States, has been an area of active investigation in the epidemiological community for nearly a decade; we refer the reader to recent articles [15, 16, 17, 18, 19] and the references therein. Contests like the CDC Forecasting Challenge [20, 15] have fostered innovation and constant information exchange among the researchers in the field. [21] and [22] provide extensive reviews on the different approaches and methodologies in practice for forecasting seasonal influenza. Influenza activity in the US is reported by the CDC

on a weekly basis as the percentage of visits with Influenza-Like Illness symptoms to the hospitals in an outpatient surveillance network known as the ILINet. Currently these are being reported at national, regional (Health and Human Services (HHS)) and state levels of aggregation. In order to forecast this influenza activity, researchers have used wide ranging data sources including Google search trends [23], social media [24], medical claims [25], and weather data [26]. Recently, authors in [27] adopted a metapopulation approach along with human mobility data to forecast sub-national influenza activity for 35 US states.

A recent study that combined these different facets and modeled sub-city influenza dynamics for New York City (NYC) was reported in [28]. Similar to our work, they used a metapopulation modeling approach to forecast influenza activity at the borough level (and zip code level) within NYC. The main focus of the authors was to evaluate the presence and absence of travel networks on the forecast performance. A gravity model was used for the travel network and the surveillance data used to evaluate the performance was the ILI% obtained at borough level. In contrast, in our work we test the effectiveness of different networks derived from official surveys, aggregated location history and mobility models.

Supplementary Methods

As described earlier, we use a metapopulation modeling framework to simulate the disease dynamics at the county level. In order to simulate and validate the model against observed ground truth, we need to perform the following steps:

- **Data preparation:** This pertains to preparing both the input data (mobility flows) and the ground truth data (disease surveillance) to match the requirements of the disease model.
- **Disease simulation:** The disease model is responsible for using the population and mobility information in order to generate simulated trajectories of disease evolution, which can then be calibrated to and compared against the ground truth surveillance. For this purpose, we use a metapopulation modeling framework (referred to as *PatchSim*).
- **Model calibration:** In order to generate short-term and seasonal target forecasts from the disease model, it needs to be calibrated against the ground truth surveillance. We adopt a Bayesian approach to calibrate the model and estimate the unknown parameters.

Before describing each of these steps in detail, we provide a description of the mobility map (AMM) as obtained from Google Location Services.

AMM Description

With the aim to better understand and improve infrastructure development and health factors, we use aggregate mobility data from Google Location Services. All data was anonymized and aggregated using a differentially private Laplace mechanism. The automated Laplace mechanism adds random noise drawn from a zero mean Laplace distribution and yields (ϵ, δ)-differential privacy guarantee of $\epsilon = 0.66$ and $\delta = 2.1 \times 10^{-29}$ per metric. Specifically, for each week W and each location pair (A,B), we compute the number of unique users who took a trip from location A to location B during week W. To each of these metrics, we add Laplace noise from a zero-mean distribution of scale 1/0.66. We then remove all metrics for which the noisy number of users is lower than 100, and publish the rest. This yields that each metric we publish satisfies (ϵ, δ)-differential privacy with values defined above.

The parameter ϵ controls the noise intensity in terms of its variance, while δ represents the deviation from pure ϵ-privacy. The closer they are to zero, the stronger the privacy guarantees. For example, with these values of the parameters, an attacker with perfect knowledge on all users except user U would increase the level of certainty as to whether U went from geographical area A to area B during a given week no more than 16%. Each user contributes at most one increment to each partition. If they go from a region A to another region B multiple times in the same week, they only contribute once to the aggregation count. No individual user data was ever manually inspected, only heavily aggregated flows of large populations were handled.

Opted-in devices securely send periodic location updates containing timestamped geolocation (latitude, longitude, time). The location is inferred from a variety of signals on-device including GPS, WiFi and cell tower triangulation. We first apply machine learning to the anonymized data to segment a raw GPS trace into semantic trips. The system automatically finds trips by taking into account a variety of signals, such as timing of location points, dwell times, and other factors. You can privately view your own detected trips in Google Maps, using the Timeline feature. We only consider location readings that are accurate within the given geographical area (e.g. NYC boroughs). We "snap" each reading into a spacetime bucket by discretizing time into longer intervals (e.g., weeks) and each (latitude, longitude) pair into a unique identifier of the geographical area (e.g., "Manhattan, NY") using publicly available data on region boundaries. As can be seen, aggregating into these large spacetime buckets further protects privacy. Finally, for each pair of geographical areas (a, b) and time interval t, the system computes the relative flow f_r between the areas within the interval, applies differential privacy filters, and outputs the anonymized and aggregated mobility map. The relative flow $f_r(a, b, t)$ is computed as a proportion of a given flow to the maximal flow in the map.

$$f_r(a, b, t) = \frac{f(a, b, t)}{max_{i,j,t'} f(i, j, t')}$$

The resulting map is indexed for efficient lookup as used to fuel the modeling described herein.

In this paper, we aggregate flows within the US spatially at county level and temporally at week level to obtain the *mobility map* (AMM). AMM contains normalized flows between pairs of counties in each week. The normalized flows are of the form $\frac{U_{t,ij}}{C}$, where $U_{t,ij}$ is the number of unique users making a trip from county i to county j in week t, and C is an undisclosed constant larger than the maximum flow over the entire year $C > max_{t,i,j} U_{t,ij}$. This dataset covers most counties (3099) in the US except those in Hawaii and DC. By checking the Pearson correlation between flows of different years (aligned on week, source, and destination), the flows are highly positively correlated (0.99) across different years.

Data preparation

Mobility data preparation

In this work, we construct mobility networks (i.e. commuting flows between counties) based on various mobility datasets, including AMM, the commute flow data obtained from the American Community Survey (ACS), unconstrained gravity model and radiation model. For any region R, as a subset of the US counties, e.g. New York City, we generate a directed weighted network $\mathcal{G}(V, E)$, where V represents counties of R; $E = \{e(i, j, F_{ij}) | i, j \in V\}$ is the edge set where $e(i, j, F_{ij})$ denotes the edge from i to j with edge's weight F_{ij}. Edge weight is defined as

$$F_{ij} = \frac{f_{i,j}}{\sum_{k \in V} f_{i,k}}$$

where flow $f_{i,j}$ comes from the underlying mobility model or dataset. Note that by definition $\sum_{j \in V} F_{ij} = 1$.

Based on the AMM data, commuter flow data, gravity and radiation models, we construct four mobility networks namely AMM, COMMUTE, GRAVITY, and RADIATION. (*i*) **AMM**: $f_{i,j}$ is the normalized Google mobility flows $\frac{U_{t,ij}}{C}$ averaged across weeks in the influenza season (for example, 2016-17 season spans from 2016 week 35 to 2017 week 34). (*ii*) **COMMUTE**: $f_{i,j}$ is the commuter count from county i to county j where $i \neq j$, obtained from ACS 2009-2013. In addition to the commuters within county i, the flow $f_{i,i}$ includes the non-commuting population calculated by subtracting all commuter counts from population size P_i of county i: $P_i - \sum_{j \neq i} f_{i,j}$. (*iii*) **GRAVITY**: $f_{i,j}$ is the gravity flow calculated as $\frac{P_i P_j}{(d_{ij}+1)^2}$, where P_i, P_j represent the population sizes (of year 2013 from US Census) of county i and j, and d_{ij} denotes the distance between i and j computed as the great-circle distance between the county centroids. (*iv*) **RADIATION**: Using distances and population sizes as above for GRAVITY model, and by the definition in [29], we compute the radiation model flows $f_{i,j}$ as $T_i \frac{P_i P_j}{(P_i+P_j+S_{ij})(P_i+S_{ij})}$ where $S_{ij} = \sum_{k:d_{ik} \leq d_{ij}} P_k$ is the total population in the circle centered at i and radius d_{ij}. T_i is the total commuter outflow from each patch, and is modeled as $T_i = \gamma P_i$, with $(1-\gamma)P_i$ set as the self-loop flow. For NYC and NJ experiments, based on US commuter data analysis in [29] we set $\gamma = 0.11$. These flows are then normalized to be compatible with the simulation model. The mobility networks are constructed for New York City (consisting of five counties) and a region of two states, New York plus New Jersey (consisting of 83 counties) based on the above equations.

We adopted a similar approach to obtain the COMMUTE, GRAVITY, RADIATION and AMM flows for Australia. While in NYC we simulated at the level of boroughs (counties), for Australia, we chose to simulate at the spatial scale of states, based on surveillance data availability and also to showcase the generality of the AMM dataset. Interstate commuter flows were obtained from the Australian Labor Market Statistics (cat. no. 6105.0) based on the 2006 Census data. For the RADIATION model, based on median commuter outflow ratio to population sizes, γ was set to be 0.004. Comparison of the normalized mobility flows of COMMUTE, GRAVITY and AMM for Australia is provided as part of Additional Analyses and Results.

Surveillance data preparation

The surveillance data used in this work includes:

- **ILI Emergency Department (ED) visits for NYC** provided by the NYC Department of Health. It contains daily ED visits for ILI per county within NYC for the past ten seasons. For instance, the daily ED counts are shown in Supplementary Figure 1a, where *Sim_Day* 0 corresponds to the beginning of MMWR week 40, 2016. Before using this for calibration, the daily ED visits are aggregated to weekly data and scaled by the influenza virus isolation rates (aka percent positive, provided by WHO-NREVSS) to obtain the ILI+ epicurves. We used the isolation rates corresponding to HHS Region 2, which includes NYC. The resulting ILI+ curve is shown in Supplementary Figure 1b.

- **Flu positive counts for NJ state** provided by the NJ Department of Health. It is a weekly cumulative laboratory count for ILI per county for the past three seasons from week 40 to the next year's week 20. We calculate the weekly new infected case count by subtracting the cumulative case count of the previous week from that of the current week. The 2016-2017 season is shown in Supplementary Figure 1c.

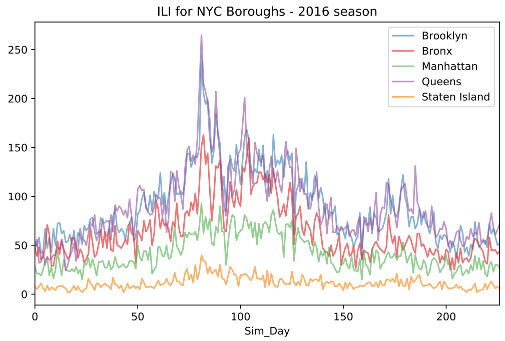

(a) NYC ILI ED visits (daily)

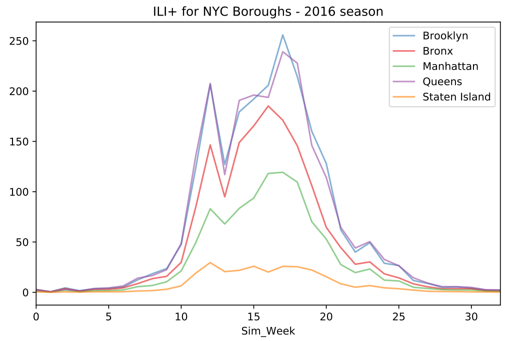

(b) NYC ILI+ ED visits (weekly)

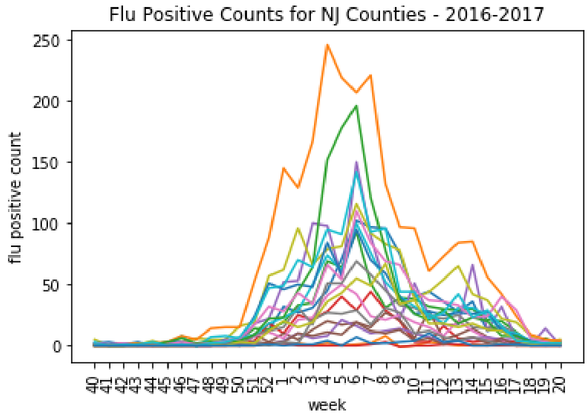

(c) NJ Flu positive counts

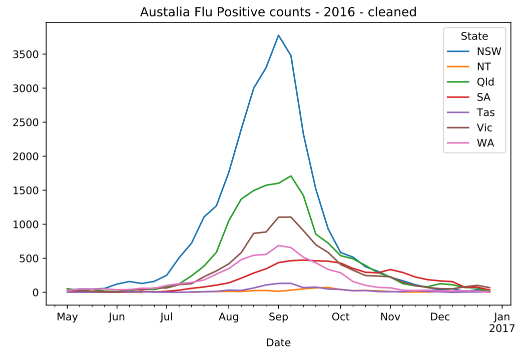

(d) NJ Flu positive counts

Supplementary Figure 1: Surveillance data. (a) ILI ED visits in NYC boroughs. *Sim_Day* denotes the days of season 2016-17 starting from the first day of epi-week 40. (b) ILI+ ED visits in NYC boroughs. *Sim_Week* denotes the epi-weeks within season 2016-17 starting from MMWR week 40. (c) Flu positive counts in NJ counties. The x-axis denotes the MMWR weeks from 2016 week 40 to 2017 week 20. (d) Lab confirmed influenza counts at state level for Australia from May to December 2016

- **ILI % for NY state and HHS2 region** provided by the Centers for Disease Control and Prevention (CDC). It is the weekly new infected case count of the past three seasons.

- **Lab confirmed influenza for Australia** Influenza surveillance data for the year of 2016 was obtained from the National Notifiable Disease Surveillance System (NNDSS) maintained by the Australian Government Department of Health (`http://www9.health.gov.au/cda/source/pub_influ.cfm`). The public dataset contains notification data collected on laboratory confirmed influenza via NNDSS at weekly resolution, for the states (excluding Australian Capital Territory), classified by type/subtype, age, sex etc. We computed the total influenza positive counts per week (Supplementary Figure 1d) from May to December 2016. Since the states differed in typical positive counts during non-influenza weeks, we removed the baseline (the minimum count for that state in the year) to obtain the ground truth for the metapopulation model.

Disease simulation

Mechanistic approaches to disease simulation often fall under one of two categories:

- **Compartmental models** Approaches of this kind are based on ordinary differential equations with the central assumption being *homogeneous mixing* of individuals within the population of interest. While easy to setup and simulate, they often cannot reproduce spatial or social heterogeneity observed in the ground truth.

- **Networked agent-based models** Approaches of this kind simulate the disease dynamics on a graph (e.g., social network) where disease propagates from infected to susceptible individuals through the edges of the graph, capturing social interactions. They are implemented in an agent-based manner and allow for high fidelity of representation. However, such models are tough to setup and pose computational challenges in model simulation and calibration.

Metapopulation models take advantage of both these approaches, and are well suited to capture spatial heterogeneity in disease dynamics. The population of interest is divided into spatially distinct *patches*, and within each patch the disease dynamics are simulated with a homogeneous mixing assumption. The patches are also connected to each other through a weighted directed network capturing movement of individuals between the patches. The movement is often representative of *commuting* (as against *migration*), thus preserving the home population counts of each patch. While within a single patch the disease evolution resembles a homogeneous compartmental model, the mobility network generates heterogeneity and longer hops between the spatial sub-populations. PatchSim is a deterministic implementation of this approach.

Let $\mathcal{N}$ represent the set of all patches (with $N = |\mathcal{N}|$). Associated with each patch i, we have population P_i, and state tuple $Z_i(t)$ denoting number of individuals in each of the disease states at time t. For a typical SEIR (Susceptible $\rightarrow$ Exposed $\rightarrow$ Infected $\rightarrow$ Recovered) model, the set of states is given by $\mathcal{Z} = \{S, E, I, R\}$. The state tuple is then $Z_i(t) = (S_i(t), E_i(t), I_i(t), R_i(t))$, with $\sum_{z \in \mathcal{Z}} z_i(t) = P_i$. Between a pair of patches i and j, we have the flow F_{ij}, denoting the fraction of individuals belonging to *home* patch i spending their day in *away* patch j. In order to conserve patch populations (i.e., commuting model), we assume $\sum_{j \in \mathcal{N}} F_{ij} = 1$. The mobility is assumed to be *homogeneous* and *memory-less*, i.e., the commuting individuals according to F_{ij} are assumed to be picked at random from the population P_i independent of their disease state, and independently for each day of the simulation. Due to the movement of individuals, the *effective* population of patches may differ from their home population P_i. This in turn also affects the state tuple Z_i. We denote the effective population as $P_i^{\texttt{eff}}$ and the effective state tuple as $Z_i^{\texttt{eff}}(t)$. Then, $P_i^{\texttt{eff}} = \sum_{k \in \mathcal{N}} F_{ki} P_i$ and $z_i^{\texttt{eff}} = \sum_{k \in \mathcal{N}} F_{ki} z_i$ for $z \in \mathcal{Z}$.

PatchSim steps through the disease simulation in daily epochs. In order to compute the change in state tuple $\Delta Z(t) = Z(t+1) - Z(t)$, it incorporates (i) movement of individuals from their respective home patches to away patches according to F_{ij}, (ii) exposures, infections, and recoveries happening in the away patches, and (iii) integration of state updates at the home patches. Let β represent the probability of exposure per day per $S - I$ contact, α the infection rate and γ recovery rate. α can be thought of as the reciprocal of mean incubation period, and γ the reciprocal of mean infectious period. Additionally, let $X_i(t)$ represent the spatio-temporal seeding profile. This captures the number of individuals of patch i who are extraneously moved from S to E to indicate external exposure ('seed' cases). The state update equations can then be written down as below for each $z \in \mathcal{Z}$ (omitting time index t for clarity):

$$\Delta S_i = -X_i - \sum_{j\in\mathcal{N}} F_{ij}\beta \frac{I_j^{\texttt{eff}}}{P_j^{\texttt{eff}}} S_i \tag{1}$$

$$\Delta E_i = X_i + \sum_{j\in\mathcal{N}} F_{ij}\beta \frac{I_j^{\texttt{eff}}}{P_j^{\texttt{eff}}} S_i - \alpha E_i \tag{2}$$

$$\Delta I_i = \alpha E_i - \gamma I_i \tag{3}$$

$$\Delta R_i = \gamma I_i \tag{4}$$

The summation in Equation 1 captures new exposures for individuals with home patch i, summed across potential away patches j. F_{ij} denotes the movement to away patch j, $\frac{I_j^{\texttt{eff}}}{P_j^{\texttt{eff}}}$ is the proportion of infectious individuals in the effective population at patch j, and β the probability of exposure given contact. Note that, unlike exposure, becoming infectious ($E \to I$) and recovery ($I \to R$) are independent of the away patch j visited by an individual, hence need not be explicitly summed across $j \in \mathcal{N}$.

Thus, given the disease parameters (β, α, γ) and a seeding profile X, PatchSim uses the population vector P and flow matrix F to produce the spatio-temporal evolution of disease states Z. In the context of the NY-NJ study, the population vector P is given by the county populations obtained from Census (for the counties in New York/New Jersey) and F is obtained from the various mobility models/data sources (GRAVITY, COMMUTE, RADIATION and AMM). The disease parameters and seeding profile X are estimated via calibration. Further, since the surveillance data is aggregated at weekly resolution, we aggregate the model output (which is daily) to match the ground truth. A scaling factor sf is also used to translate infection case counts produced by PatchSim to emergency department visits.

Model calibration

Model calibration is the process of estimating the unknown parameters of the model with the help of observed data. Here, we will estimate the disease parameters and seeding profile of our disease simulation PatchSim using observed ground truth of influenza surveillance. We adopt a Bayesian approach to calibrate the PatchSim model, where we begin with a prior distribution on the unknown parameters, which are then combined with the data likelihood to produce the posterior distribution on the parameter space. Inferences on parameters are carried out by sampling from the posterior. We first describe the general framework below.

We define a statistical model for the observed data as a noisy version of model output (in this case, PatchSim). Let

$$y_i(t) = \eta_i(t,\theta) + \epsilon_i(t), \qquad i \in \mathcal{N}, t \in [1,T] \tag{5}$$

where $y_i(t)$ is the ground truth data for patch i at time t, $\eta_i(t,\theta)$ is the corresponding PatchSim output given model parameters θ and $\epsilon_i(t) \overset{iid}{\sim} N(0,\sigma^2)$. T is the *data horizon*, i.e., the number of time steps in the observed ground truth. Assuming independent error terms across (i,t), the likelihood of observing the ground truth, given the PatchSim with parameter θ can be written as:

$$L(y|\theta) = \frac{1}{(\sqrt{2\pi}\sigma)^{|\mathcal{N}|T}} \prod_{i\in|\mathcal{N}|} \prod_{t\in[0,T]} \exp\Big\{ -\frac{(y_i(t)-\eta_i(t,\theta))^2}{2\sigma^2} \Big\} \tag{6}$$

Given the prior distribution $\pi(\theta)$ and the data likelihood $L(y|\theta)$, the posterior distribution can be written as:

$$\pi(\theta|y) = \frac{L(y|\theta)\pi(\theta)}{\pi(y)} \propto L(y|\theta)\pi(\theta)$$

where, $\pi(y) = \int_{\boldsymbol{\theta}} L(y|\theta)\pi(\theta)\, d\theta$, is the normalization constant.

A brief primer on importance sampling: The posterior distribution often does not belong to known family of distributions, because of the complex simulation model η. Monte Carlo approaches to explore the posterior space are used in such situations. Importance sampling is one such technique to generate realizations from a given distribution. For instance, given a probability density function p on $\Omega \in \mathbb{R}^d$ to compute the expectation $\mu = E_p(f(X))$, we can rewrite it as

$$E(f(X)) = \int_\Omega f(x)p(x)dx = \int_\Omega \frac{f(x)p(x)}{q(x)}\, q(x)dx = E_q\left(\frac{f(x)p(x)}{q(x)}\right)$$

where, E_q denotes the expectation with respect to the probability density function q, defined on $\mathbb{R}^d$. q is called an importance distribution, which is usually a known distribution, easy to sample from, and the ratio $p(x)/q(x)$ is known as the importance ratio, which accounts for the adjustment since x's are sampled from q rather than from p. A Monte Carlo estimate of this expectation can then be computed by sampling $x_1, \cdots, x_n$ from q and by calculating

$$\hat{\mu} = \frac{1}{n}\sum_{i=1}^{n} \frac{f(x_i)p(x_i)}{q(x_i)}.$$

Similarly any such random sample $x_1, \cdots, x_n$ from q along with their normalized importance weights $w_i = p(x_i)/q(x_i)$ (so that $\sum_i w_i = 1$) provides a direct approximation of p by,

$$\hat{p} = \sum_{i=1}^{n} w_i \delta(x_i).$$

where $\delta(x_i)$ is a Dirac delta function at x_i. We use importance sampling to sample from out posterior distribution $\pi(\theta|y)$ (corresponding to $p(x)$ above). Our choice of importance distribution (corresponding to $q(x)$ above) is the prior $\pi(\theta)$. This reduces the calculation of importance weights to just computing the data likelihood L at each sample from the prior, i.e.

$$w_i = \frac{\pi(\theta_i|y)}{\pi(\theta_i)} = \frac{L(y|\theta_i)\pi(\theta_i)}{\pi(\theta_i)} = L(y|\theta_i). \tag{7}$$

A re-sample $\{\hat{\theta}_1, \cdots, \hat{\theta}_m\}$ from $\{\theta_1, \cdots, \theta_n\}$ with probabilities proportional to $\{w_1, \cdots, w_n\}$, with replacement constitutes a sample of size m from the posterior $\pi(\theta|y)$. The calibrated forecast can then be produced by running the PatchSim model at the parameter values $\hat{\theta}_i$, which produces m time series, which are then used to compute other summary statistics on the forecast.

In our context, in addition to the disease parameters β, α, and γ, we also want to estimate the seeding profile. However X is $|\mathcal{N}| \times T$-dimensional, and is prohibitively expensive to calibrate. Hence, we parameterize the seeding using two additional parameters *seedT* and *seedN*. *seedT* represents the noise level in the ground truth above which the seeding is assumed to occur, and *seedN* controls the number of patches in which the seeding is done. For instance if $seedT = 5$ and $seedN = 3$, we first identify the weeks W_i at which each of the counties exceed five ED visits. We identify the three earliest counties to reach the threshold (least W_i) and seed them respectively

Supplementary Table 2: Parameter ranges

Parameter	Range
Exposure Rate (β)	[0.5,0.9]
Infection Rate (α)	[0.5,0.9]
Recovery Rate (γ)	[0.5,0.9]
Seeding Threshold ($seedT$)	[5,25]
Seeding Patch Count ($seedN$)	[1,5]
Scaling Factor (sf)	[0.001, 0.005]

on week W_i with count $ED(W_i)$. The complete set of parameters with their ranges are given in Table 2. With slight abuse of notation, we use θ to denote all the parameters being calibrated, including $seedT$, $seedN$, and sf with the SEIR parameters β, α, and γ.

We generated a full-factorial design Θ on these parameters, with 10 levels each for disease parameters and 5 levels each for other parameters (125000 experiment cells in all), assuming discrete uniform prior $\pi(\theta)$ over the parameter space. Note that, any other suitable distribution instead of discrete uniform can be used which may reflect prior knowledge on the parameters. These are taken to be samples from the *importance distribution* and the normalized *importance weight* is computed as:

$$w(\theta) = \frac{L(y|\theta)}{\sum_{\theta \in \Theta} L(y|\theta)}, \qquad \theta \in \Theta$$

A re-sample from Θ with corresponding weights w is then considered as the sample from the target posterior distribution.

We observed that the per capita emergency department visit rate varied significantly across the counties in NYC. In order to account for this, we computed $ED_{frac}(i)$ as follows:

$$ED_{frac}(i) = \frac{ED_i/Pop_i}{ED_{NYC}/Pop_{NYC}}$$

Table 3 shows the ED_{frac} for the past three influenza seasons. The reciprocal of this value is used to scale the ED visit counts in each of the boroughs to eliminate the bias for calibration.

Supplementary Table 3: Normalized per capita ED visits (ED_{frac})

Borough	2015-16	2016-17	2017-18
Bronx	1.3623	1.2838	1.3607
Brooklyn	0.8526	0.9814	0.9137
Manhattan	0.6939	0.7083	0.7290
Queens	1.221	1.1179	1.133
Staten Island	0.7046	0.6862	0.6791

Parameters for Australia: While the calibration methodology remained the same for the Australia forecasts, we modified the parameter ranges to account for variations in disease intensity, spatial heterogeneity and surveillance data. Further, the equivalent of the normalized per capita ED visits (used as an additional data-driven scaling factor per patch in NYC) was not stable across the past three years. Hence in this case, we calibrated the scaling factor for each patch separately. Also given the increased number of parameters, we used a uniform sampling of 100000 configurations from the parameter space.

Supplementary Table 4: Parameter ranges for Australia

Parameter	Range
Exposure Rate (β)	[0.3,0.9]
Infection Rate (α)	[0.3,0.9]
Recovery Rate (γ)	[0.3,0.9]
Seeding Threshold ($seedT$)	[10,100]
Seeding Patch Count ($seedN$)	[6,8]
Scaling Factor (sf) per patch	[0.01, 0.02]

Supplementary Table 5: Network structures

**Network**	**Nodes**	**Edges**	**Density**	**Average clustering coefficient**	**Average shortest path length**
AMM	83	2634	0.39	0.0138	213
COMMUTE	83	2990	0.44	0.0041	293
GRAVITY	83	6806	1.00	0.0053	2649
RADIATION	83	6806	1.00	0.0017	539

Supplementary Notes 2

In this section we present additional analyses and results for the different mobility networks and subsequently influenza forecasting performance across seasons.

Exploratory data analysis of mobility networks

Influenza spreads from an infected individual to a susceptible individual. As a result, social proximity networks play an important role in disease transmission. Thus a realistic representation of human mobility and their human interactions are likely to to improve the quality of mathematical models for forecasting ILI dynamics.

To further explore the four mobility networks generated in this paper, we compare their structural features with the NY-NJ networks as an example. To eliminate the effect of large self-loop flows during the comparison, we removed self-loops from the three networks. In Supplementary Table 5, we show the basic network features. Among these features, the density and average shortest path length are computed on the weighted directed networks, while the average clustering coefficient is computed on weighted undirected networks where the weight of an edge between two nodes is the summation of the weights of two directional edges. We can observe that the densities of AMM and COMMUTE networks are about 0.4. The average clustering coefficient of AMM is much larger than that of COMMUTE, GRAVITY and RADIATION, which indicates that AMM has greater local connectivity among people than the other mobility networks. In addition, AMM shows the smallest average shortest path length (with distance $dist(i, j) = 1/w(i, j)$) among the networks, which indicates that AMM has stronger global connectivity than the other two. During an epidemic transmission a stronger connectivity among regions may lead to faster and wider spread of the disease.

In Supplementary Figure 2, we show the adjacency matrices of the different networks as heat

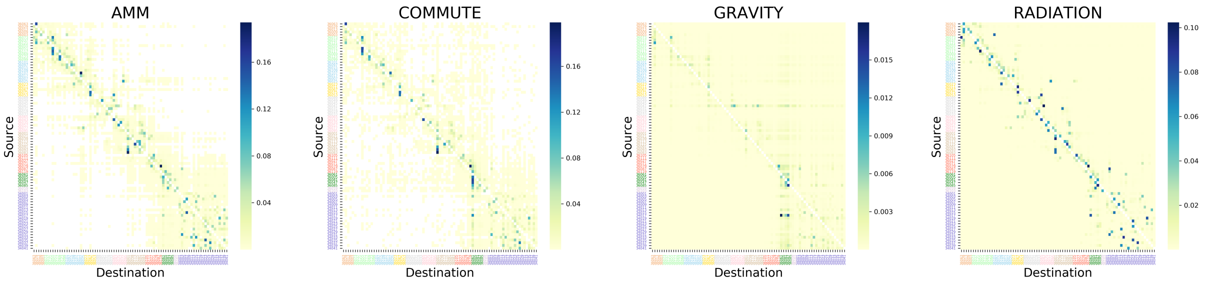

(a) AMM (b) COMMUTE (c) GRAVITY (d) RADIATION

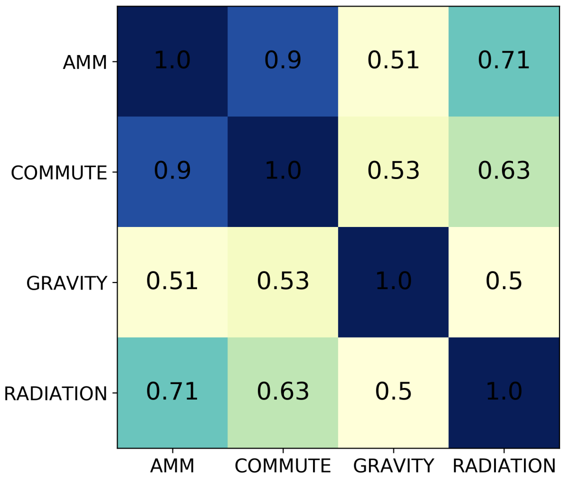

(e) correlation

Supplementary Figure 2: Adjacency matrix. Color represents $w(i, j)$ (darker color denotes larger edge weight). The counties are arranged according to spatial neighborhood. The flows are aligned by source and destination nodes. AMMand COMMUTE have highly positive correlation value (0.90), followed by RADIATION model (0.71 with AMM).

maps. From the figure we can observe that the networks are asymmetric, i.e. the flow from county i to county j is not equal to the flow from county j to county i. We also observe that while AMMand COMMUTE are sparser and seem to be clustered, GRAVITY network, by virtue of its definition, is more homogeneous. RADIATION model seems to be dense, but captures some of the local neighborhood patterns visible in COMMUTE or AMM. The correlations between networks (Supplementary Figure 2e) show that AMMand COMMUTE are highly positively correlated, followed by the RADIATION model while GRAVITY has relatively small positive correlation with the other networks.

In Supplementary Figure 5, we show a histogram of incoming flow (sum of weights of all incoming edges to county i except the self-loop $\sum_{j,i,j\neq i} F_{ji}$) and outgoing flow (sum of weights of all outgoing edges from county i except the self-loop $\sum_{i,j,j\neq i} F_{ij}$) for each county, respectively. The **x**-axis denotes incoming (outgoing) flows. The **y**-axis denotes frequency. The red dotted curves are estimated probability density functions, fitted and chosen as follows. We fit the flow data to several reference distributions, including exponential, normal, exponential power, gamma, and log-normal distributions. Then we run the KS-test (the null hypothesis being that the sample is drawn from the reference distribution) to choose a distribution with the highest significance.

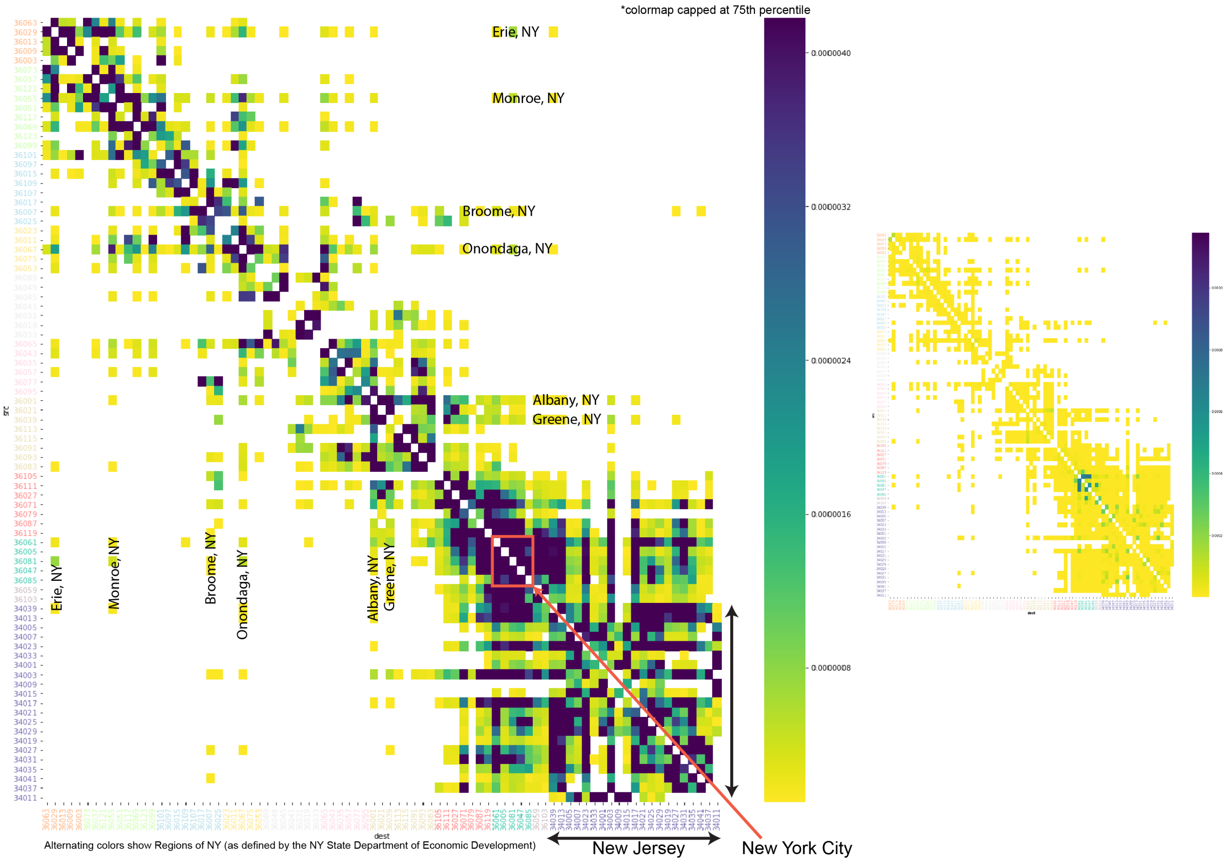

Supplementary Figure 3: Adjacency matrix of AMM with the counties of NY arranged according to spatial neighborhood. We use the Regions of New York as defined by the New York State Department of Economic Development [30]. We use the first week of 2017 as an example, since there are no qualitative visual differences across the weeks. Also note that the visualization shows the raw flows, instead of the normalized flows shown in 2. The self loop flows are not shown, since they're an order of magnitude larger than the rest. In the bigger image the colormap is capped at 75th percentile, and the smaller inset shows the full colormap where the counties of NYC dominate the others. The heatmap shows significant clustering within regions, with a few counties well connected to parts of New York City and New Jersey. Also, New York City, Hudson valley and Long Island in New York are well connected to New Jersey, due to spatial proximity.

The scale and location together with the significant value are shown in the plots. The major observations are: (i) all incoming flows follow exponential distribution, (ii) AMM, COMMUTE and RADIATION flows follow normal distribution whereas GRAVITY follows exponential distribution. According to the observations from Supplementary Figure 2 and 5, we would expect that the epidemic simulations based on AMM COMMUTE and RADIATION show greater similarity than that based on GRAVITY, which matches our forecasting performance results.

Among the centrality measures, such as degree, closeness, and betweenness, assesses the degree to which a node lies on the shortest path between any two other nodes, and is able to funnel the flow in the network. During an epidemic, a county with high betweenness probably plays a crucial role of disease spread. Controlling the infections of the county can effectively reduce the spread of the disease. Thus, whether the nodes with high betweenness in a mobility network are realistic in

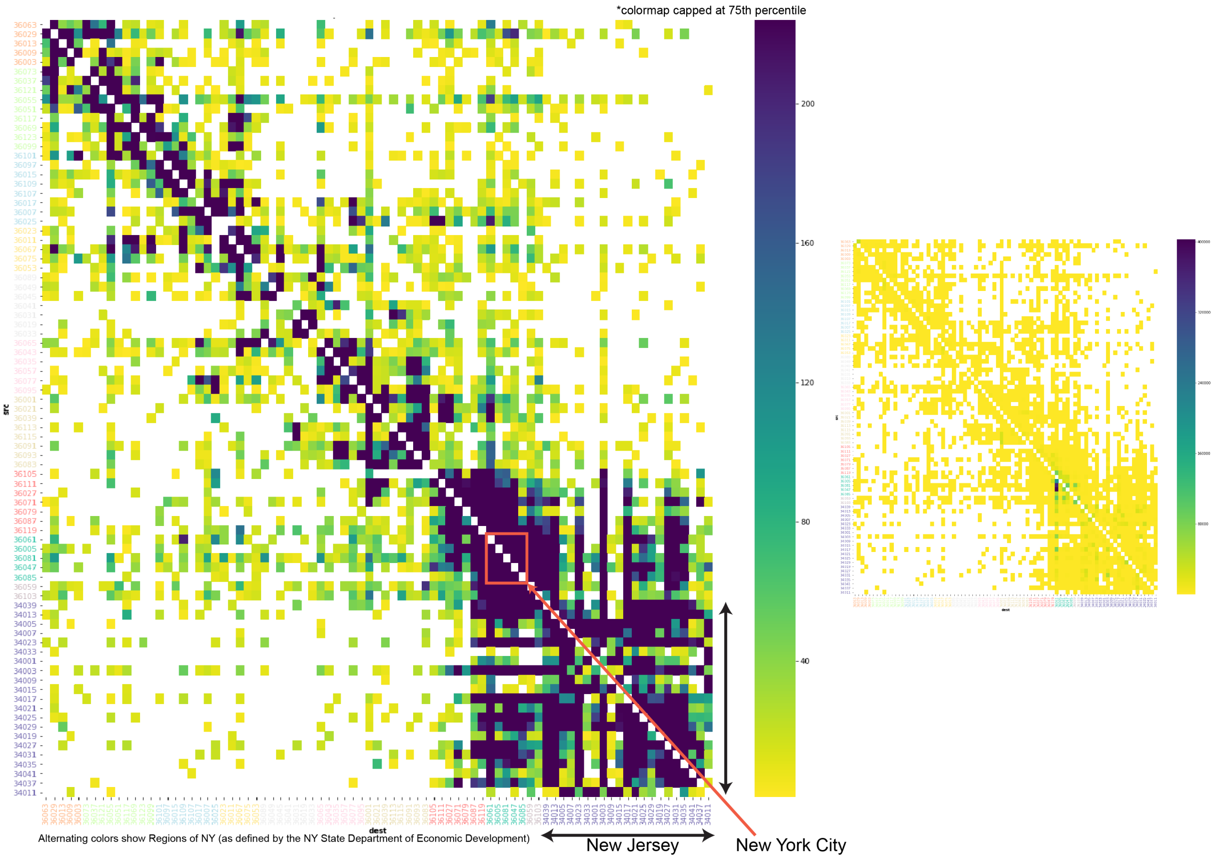

Supplementary Figure 4: Adjacency matrix of Commute flows with the counties of NY arranged according to spatial neighborhood. We use the Regions of New York as defined by the New York State Department of Economic Development [30]. Also note that the visualization shows the raw flows, instead of the normalized flows shown in 2. The self loop flows are not shown, since they're an order of magnitude larger than the rest. In the bigger image the colormap is capped at 75th percentile, and the smaller inset shows the full colormap where the counties of NYC dominate the others. Unlike the 3, we see higher number of inter-regional flows, going beyond spatial neighborhood. We can especially see significant number of commute from almost all counties in NY and NJ to the New York City.

real world scenarios is crucial for a realistic simulation based on the network.

Supplementary Figure 6 shows the county betweenness of three networks, and Supplementary Table 6 displays the top 5 counties of the highest betweenness. Firstly, the networks show different structures in terms of betweenness. While AMM and RADIATION show similar counties with top betweenness, COMMUTE picks distinct but neighboring counties. These counties connect rural counties, e.g. Allegany, NY or St. Lawrence, NY or Cape May, NJ, to highly urbanized counties like Manhattan or Brooklyn. GRAVITY model does not show any significant patterns in the top betweenness counties. These observations indicate that AMMcan capture some realistic geographical characteristics of people's mobility behavior related to the traffic and urbanization of the counties. As discussed, these realistic characteristics are crucial for better epidemic forecasting of influenza.

Gravity model calibration: In the main paper, we use an uncalibrated gravity model with

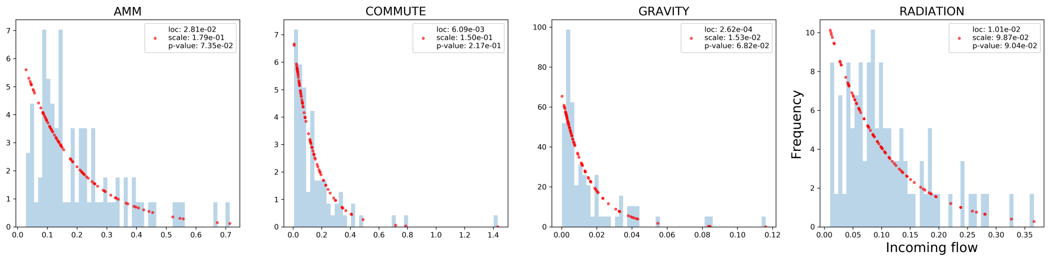

(a) Incoming flow distribution

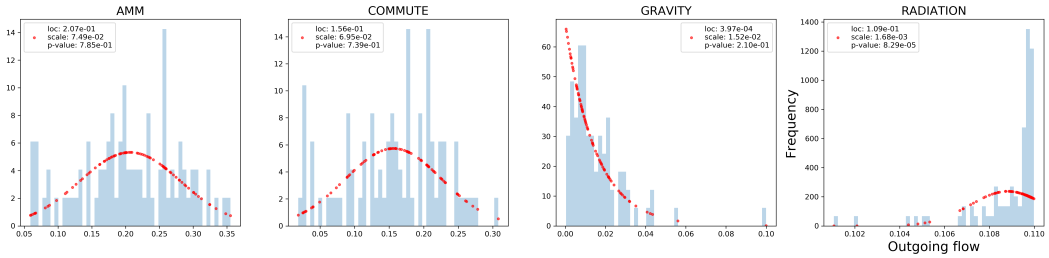

(b) Outgoing flow distribution

Supplementary Figure 5: Flow distributions. The **x**-axis denotes incoming flow (sum of weights of all incoming edges to county i except the self-loop $\sum_{j,i,j\neq i} F_{ji}$) and outgoing flow (sum of weights of all outgoing edges from county i except the self-loop $\sum_{i,j,j\neq i} F_{ij}$) for each county, respectively. The **y**-axis denotes frequency. The red dotted curves are estimated probability density functions. The scale and location together with the significant value are shown in legends. (a) Incoming flow distribution. All three flows follow exponential distributions. (b) Outgoing flow distributions of AMM COMMUTE and RADIATION are similar.

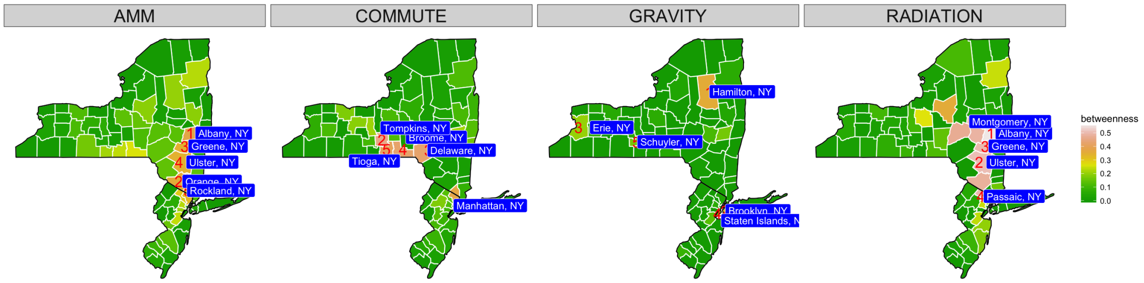

Supplementary Figure 6: Structural measures. Node betweenness centrality of different counties for the AMM, COMMUTE, GRAVITY, RADIATION networks. Note that AMM and Radiation detect similar counties as having high betweenness centrality.

distance exponent of 2, for forecast performance comparison with the COMMUTE and AMM. As mentioned earlier, this is to ensure that the networks being compared are generated independent of each other. In Supplementary Table 7, we report the coefficients estimated using a generalized linear model fitted to the COMMUTE dataset using a single parameter Poisson model as below:

Supplementary Table 6: The top 5 counties with highest betweenness in the NY-NJ networks

AMM	COMMUTE	GRAVITY	RADIATION
36001 Albany, NY<br>36071 Orange, NY<br>36039 Greene, NY<br>36111 Ulster, NY<br>36087 Rockland, NY	36061 Manhattan, NY<br>36109 Tompkins, NY<br>36025 Delaware, NY<br>36007 Broome, NY<br>36107 Tioga, NY	36041 Hamilton, NY<br>36047 Brooklyn, NY<br>36029 Erie, NY<br>36085 Staten Islands, NY<br>36097 Schuyler, NY	36001 Albany, NY<br>36111 Ulster, NY<br>36039 Greene, NY<br>34031 Passaic, NJ<br>36057 Montgomery, NY

Supplementary Table 7: Coefficients for the gravity model fitted to the COMMUTE dataset for counties in New York and New Jersey (n=130400) obtained via Generalized Linear Model Regression along with the z-values (Wald statistic for the two-sided test for the hypothesis that the estimate is zero).

	coef	std err	z	$P > \|z\|$	[0.025	0.975]
Intercept	-0.8241	0.002	-463.264	0.000	-0.828	-0.821
$\log O_i$	0.4581	0.000	3823.140	0.000	0.458	0.458
$\log D_j$	0.7334	0.000	5707.439	0.000	0.733	0.734
$\log d_{ij}$	-1.8384	0.000	$-1.13e04$	0.000	-1.839	-1.838

$$log(F_{ij}) = \beta_0 + \beta_1 * \log O_i + \beta_2 * \log D_j + \beta_3 * \log d_{ij}$$

where O_i, D_j are the population sizes of origin and destination patches, and d_{ij} is the distance between the patches (ignoring self-loop flows).

Stationarity of AMM: Although there are temporal variations in the flows (as shown in Supplementary Figure 7(a)), the autocorrelation plots are relatively flat through the entire duration (Supplementary Figures 7(b)&(c)). We only show the self-loop flows, although similar conclusions hold for each entry of the flow matrix. We also use only the normalized versions of the flows since they serve as inputs to the simulation model. The observed stationarity motivates the use of annual average of the normalized AMMflows as input to the metapopulation model.

Mobility data comparison for Australia: Supplementary Figure 8 shows the different mobility networks used for Australia. The values reported are the logarithm of the normalized flows between a pair of states. Given the sparsity of the population and coarseness of the spatial resolution, we see that all patches have high self-loop flows. For the non self-loop flows, we see that there higher similarity in magnitude and distribution between COMMUTE and AMM.

Calibration results

We tested our modeling and calibration approach on three models based on the AMM, COMMUTE, GRAVITY and RADIATION networks described in the Supplementary Methods for NYC. A comparison is also done between models using mobility data (the aforementioned four networks) and a model without mobility, calling it as *Baseline* model. To compare among these five different network models, we consider 4 week look ahead median predictions for each of the five counties.

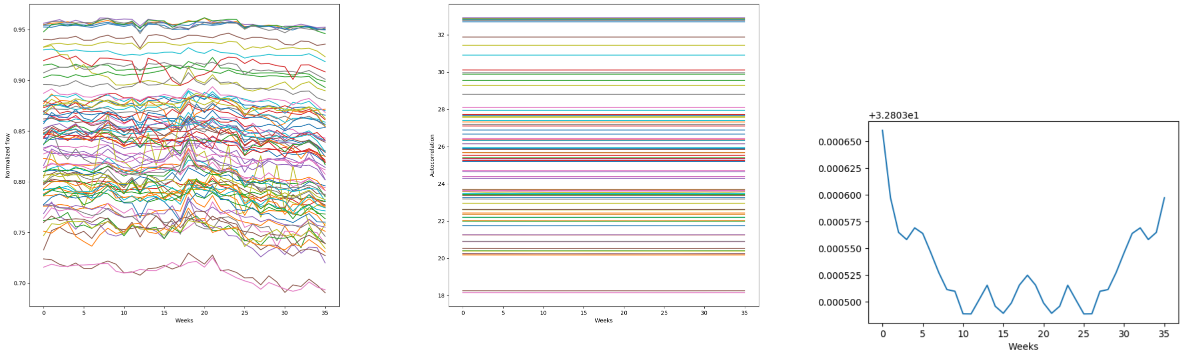

(a) (b) (c)

Supplementary Figure 7: Temporal variations in normalized AMM in NY-NJ network. (a) Normalized self-loop flows. (b)Autocorrelation of self-loop flows. (c) Sample autocorrelation plot for one of the counties.

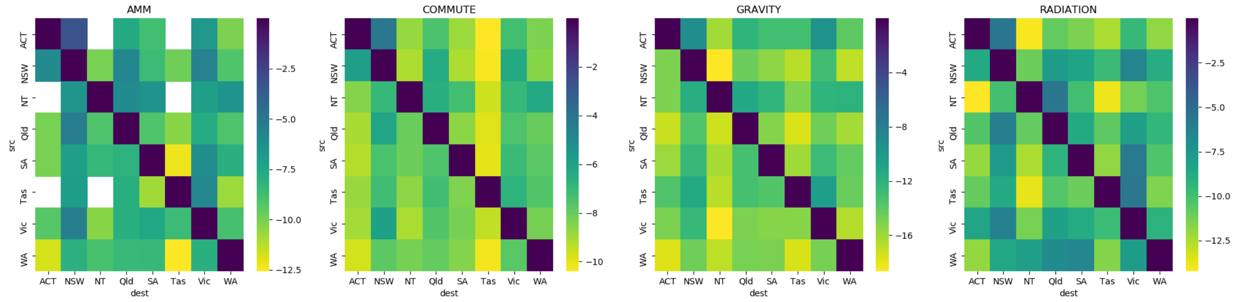

Supplementary Figure 8: Normalized mobility flows of AMM COMMUTE, GRAVITY and RADIATION for Australia.

The overall mean absolute percentage error (MAPE) for 4 week look ahead prediction is defined as

$$\frac{1}{5}\sum_{s=1}^{5}\frac{1}{4}\sum_{t=1}^{4}\left|\frac{y_{st}-\hat{y}_{st}}{y_{st}}\right|$$

We also provide the model performances in estimating the seasonal targets. i.e., peak time, peak size and onset time for each of the five boroughs.

Season 2015-2016

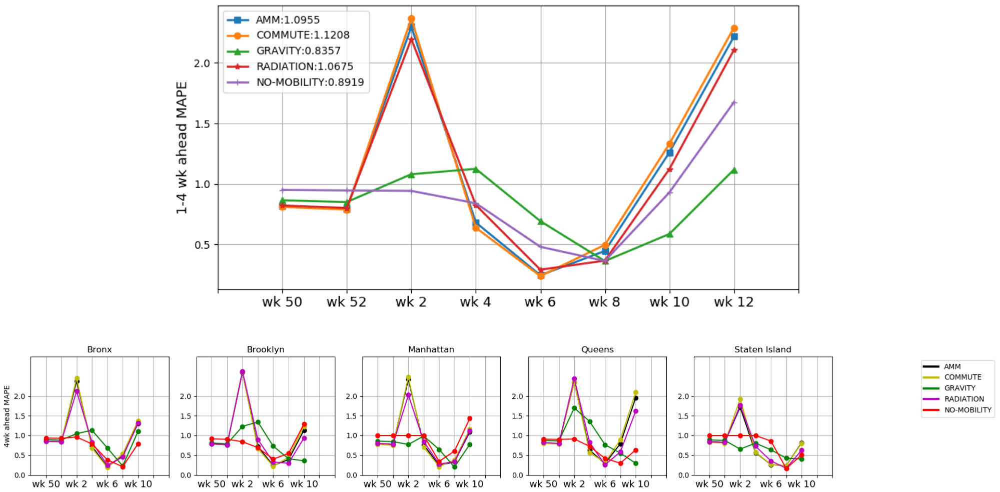

Supplementary Figure 9: Top: Mean absolute prediction error (MAPE) for 4 week look ahead prediction, averaging over 4 weeks and 5 boroughs at prediction horizon from week 50 of 2015 to week 12 of 2016 for NYC. Bottom: MAPE for 4 week look ahead prediction for each borough separately (NYC, 2015-16 season).

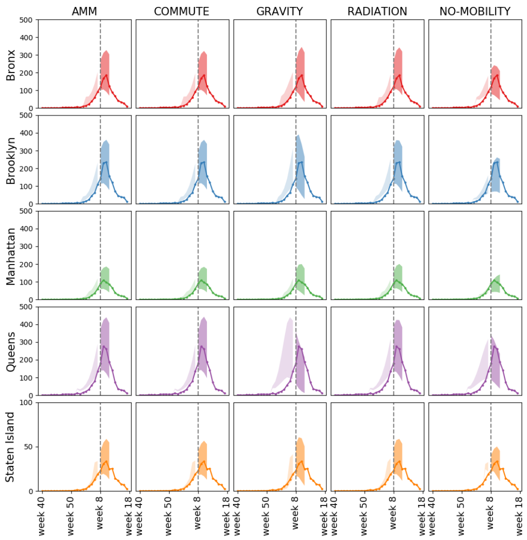

(a) Calibration and prediction at pre-peak

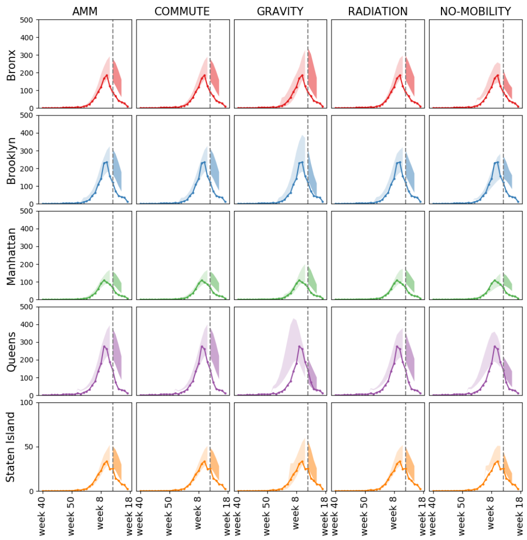

(b) Calibration and prediction at post-peak

Supplementary Figure 10: Pre and post peak 4 week ahead 90% prediction interval for the 5 models, NYC 2015-16 season.

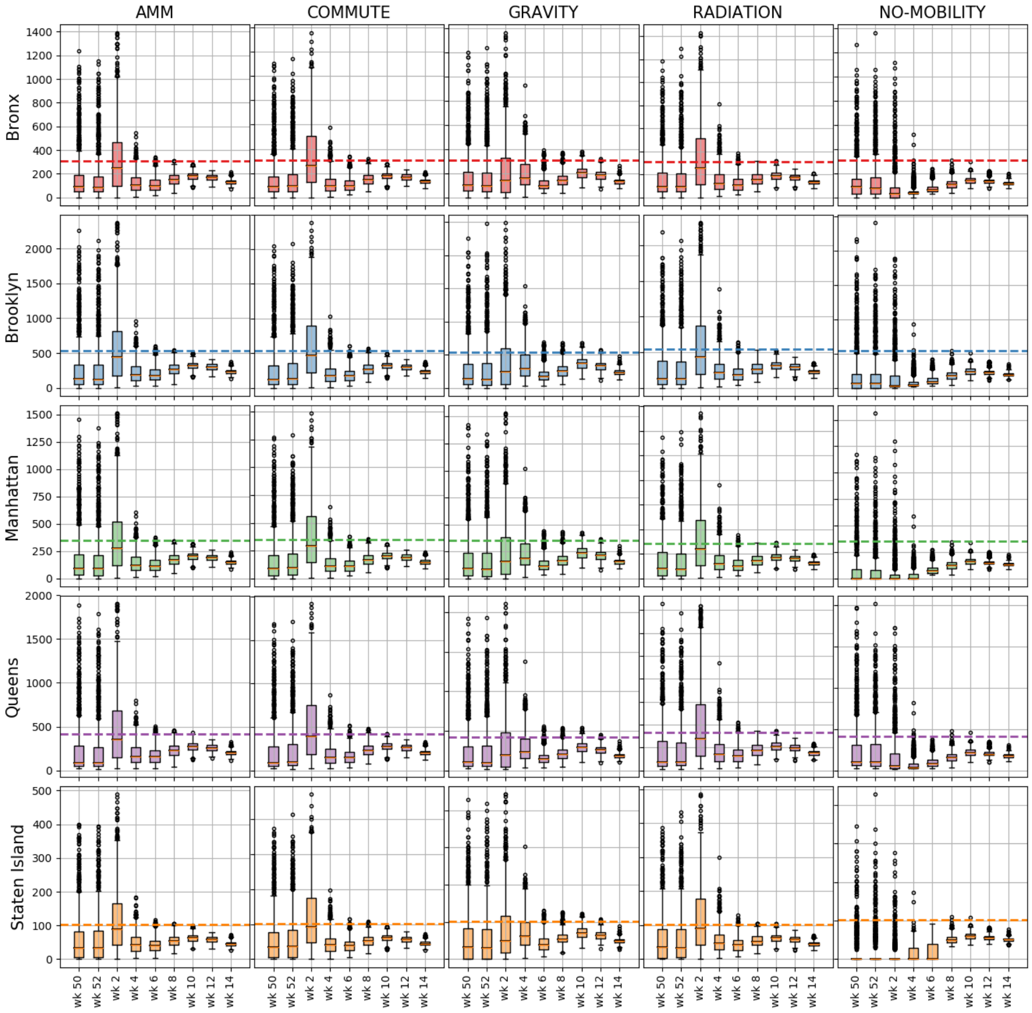

Supplementary Figure 11: The boxplots (median, IQR and whiskers at 1.5IQR) show the posterior prediction of peak intensity for 5 boroughs from 5 models at different prediction horizons (n=1000). The horizontal dashed line represents the ground truth. (NYC, 2015-16 season)

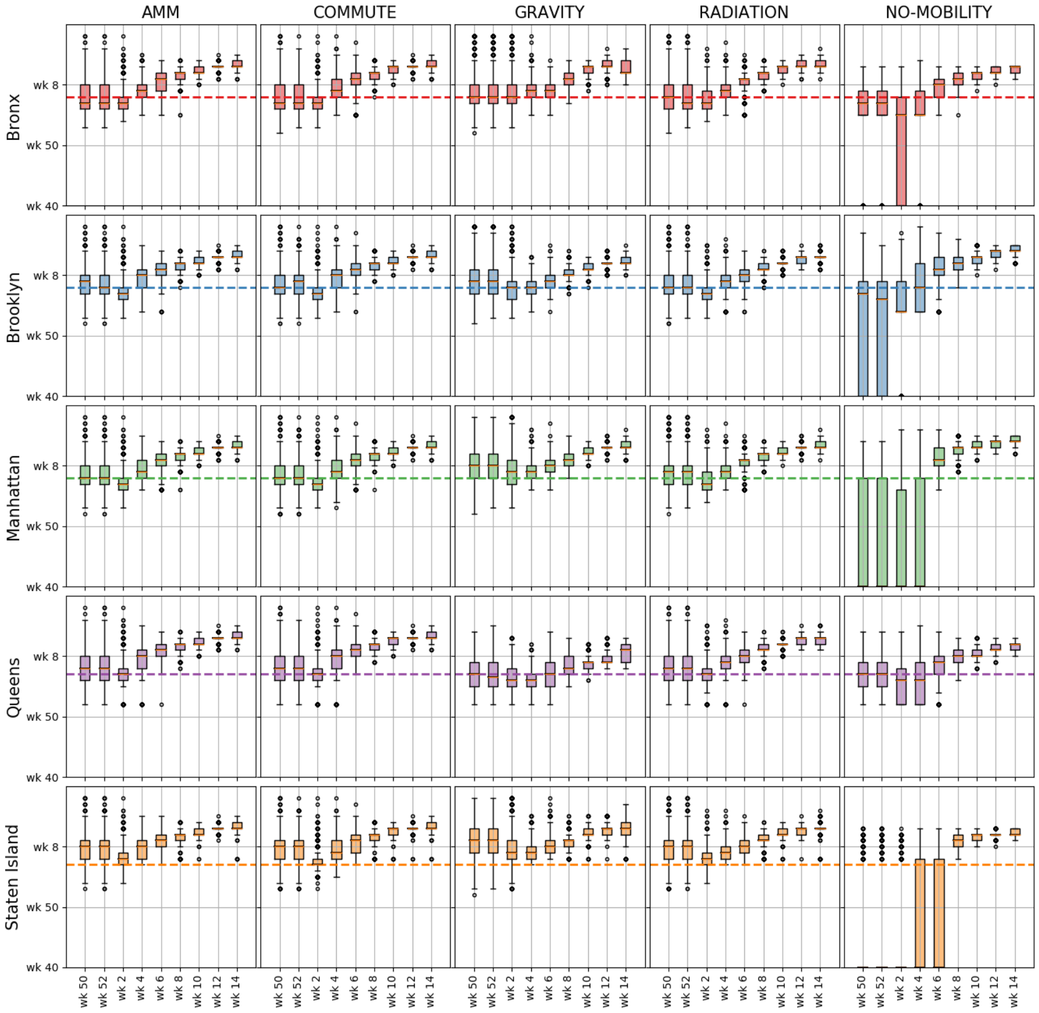

Supplementary Figure 12: The boxplots (median, IQR and whiskers at 1.5IQR) show the posterior prediction of time to peak for 5 boroughs from 5 models at different prediction horizons (n=1000). The horizontal dashed line represents the ground truth. (NYC, 2015-16 season)

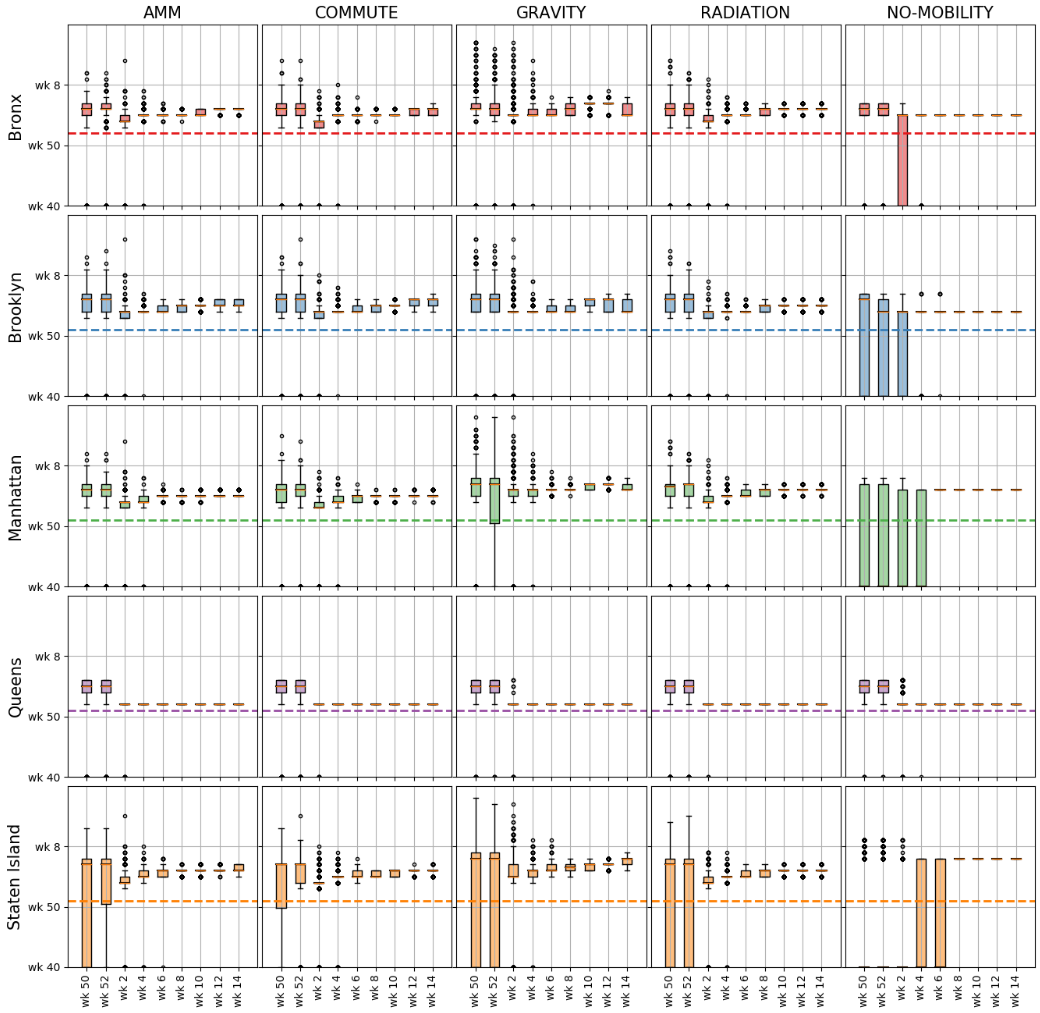

Supplementary Figure 13: The boxplots (median, IQR and whiskers at 1.5IQR) show the posterior prediction of time to onset (we defined onset to be 10% of the peak intensity) for 5 boroughs from 5 models at different prediction horizons (n=1000). The horizontal dashed line represents the ground truth. (NYC, 2015-16 season)

Season 2016-2017

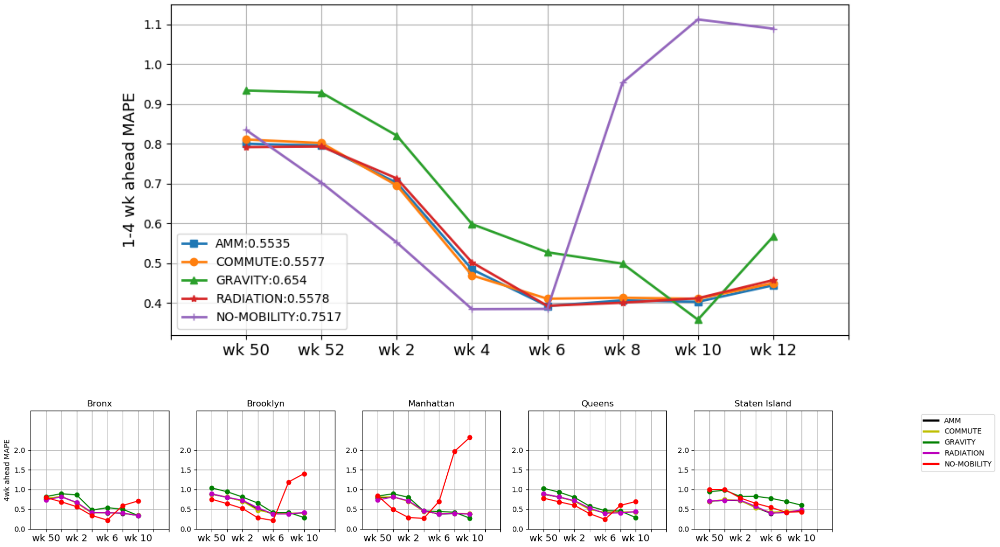

Supplementary Figure 14: Top: Mean absolute prediction error (MAPE) for 4 week look ahead prediction, averaging over 4 weeks and 5 boroughs at prediction horizon from week 50 of 2016 to week 12 of 2017 (NYC). Bottom: MAPE for 4 week look ahead prediction for each borough separately (NYC, 2016-17 season).

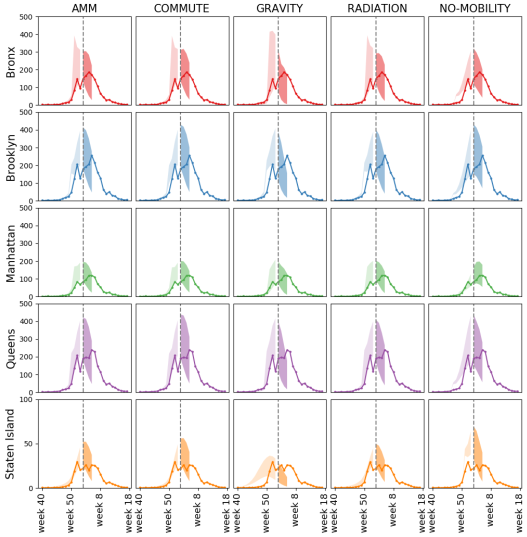

(a) Calibration and prediction at pre-peak

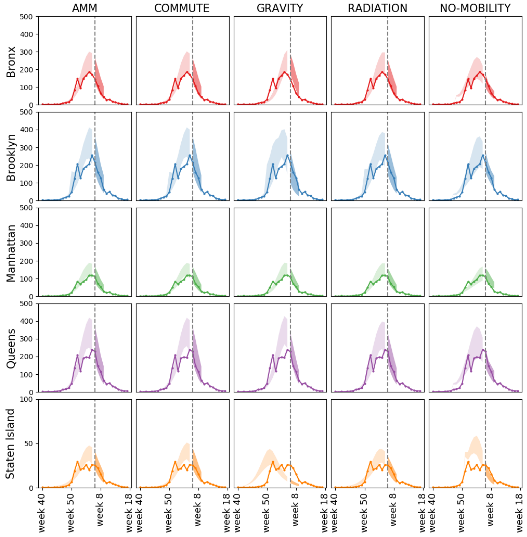

(b) Calibration and prediction at post-peak

Supplementary Figure 15: Pre and post peak 4 week ahead 90% prediction interval for 5 models (NYC, 2016-17 season).

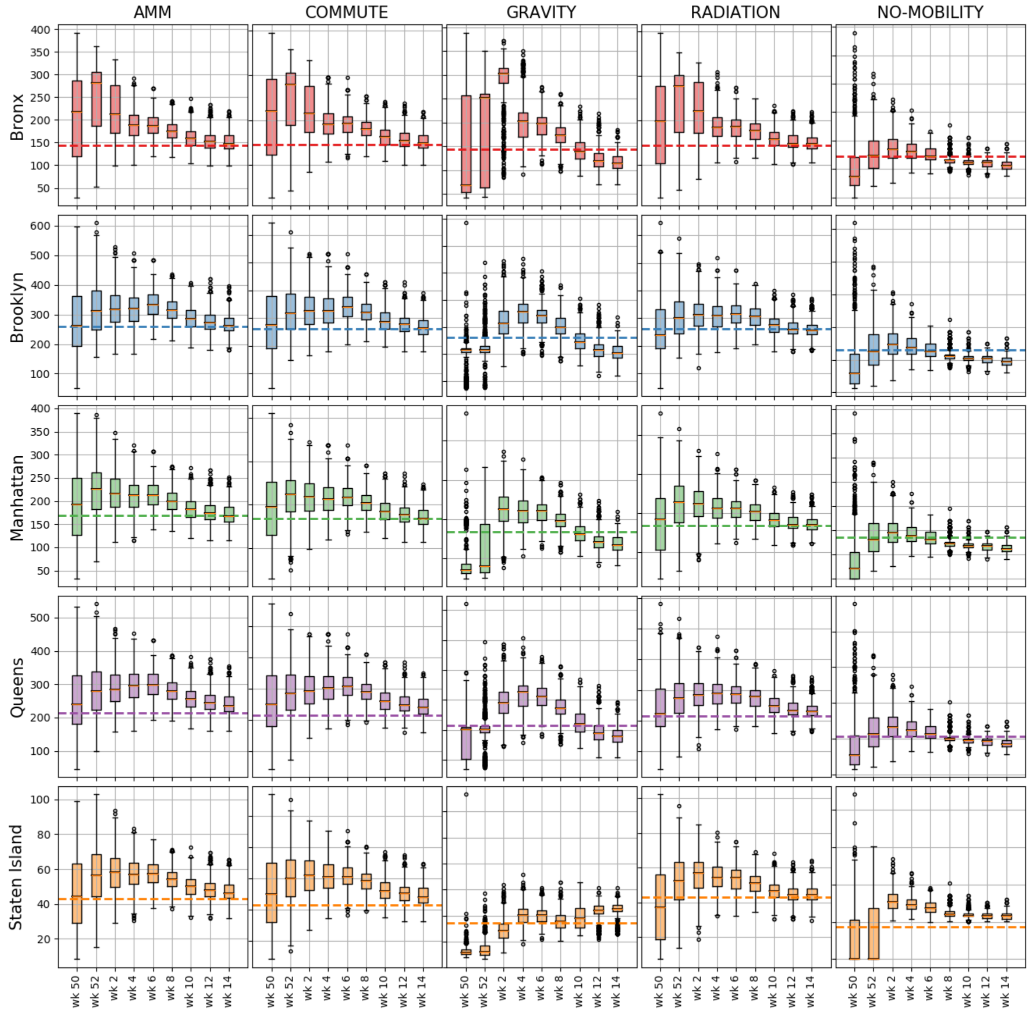

Supplementary Figure 16: The boxplots (median, IQR and whiskers at 1.5IQR) show the posterior prediction of peak intensity for 5 boroughs from 5 models at different prediction horizons (n=1000). The horizontal dashed line represents the ground truth (NYC, 2016-17 season).

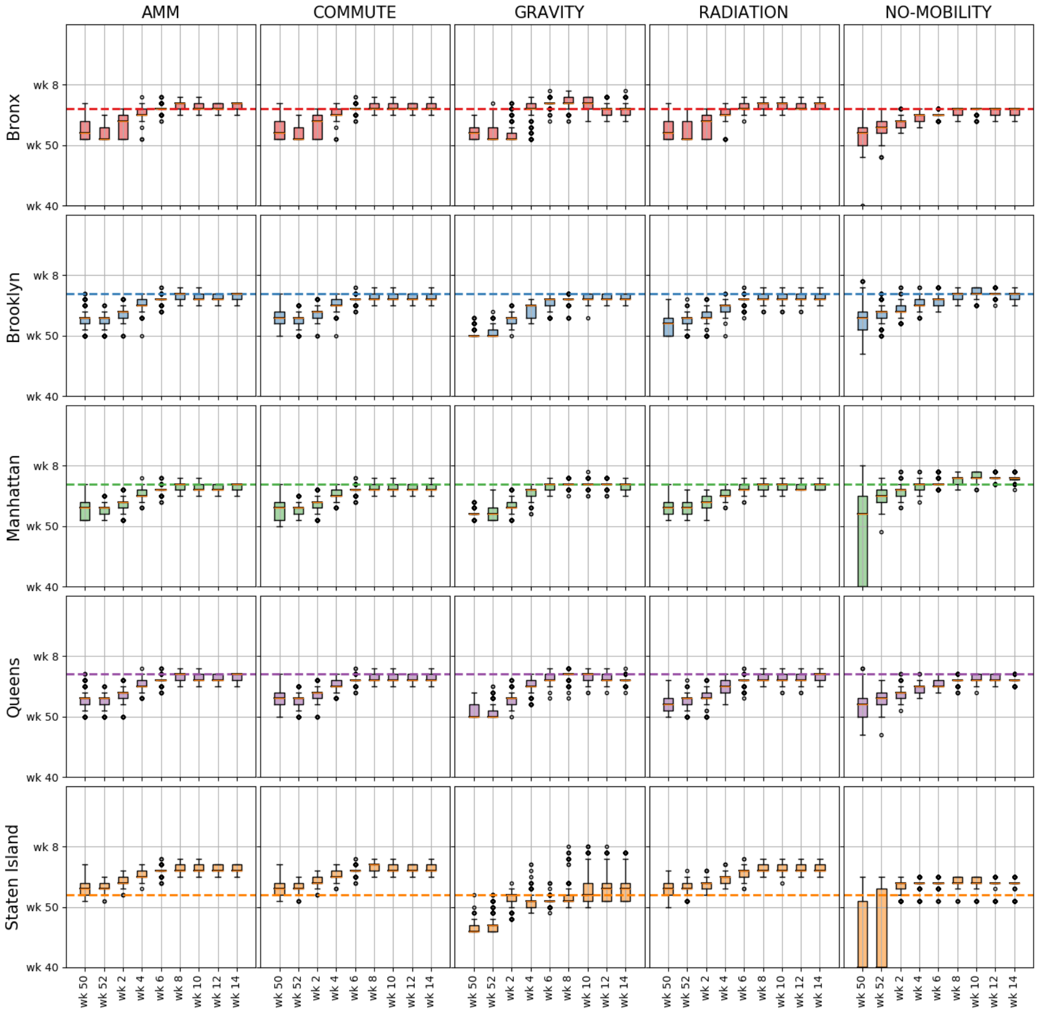

Supplementary Figure 17: The boxplots (median, IQR and whiskers at 1.5IQR) show the posterior prediction of time to peak for 5 boroughs from 5 models at different prediction horizons (n=1000). The horizontal dashed line represents the ground truth (NYC, 2016-17 season).

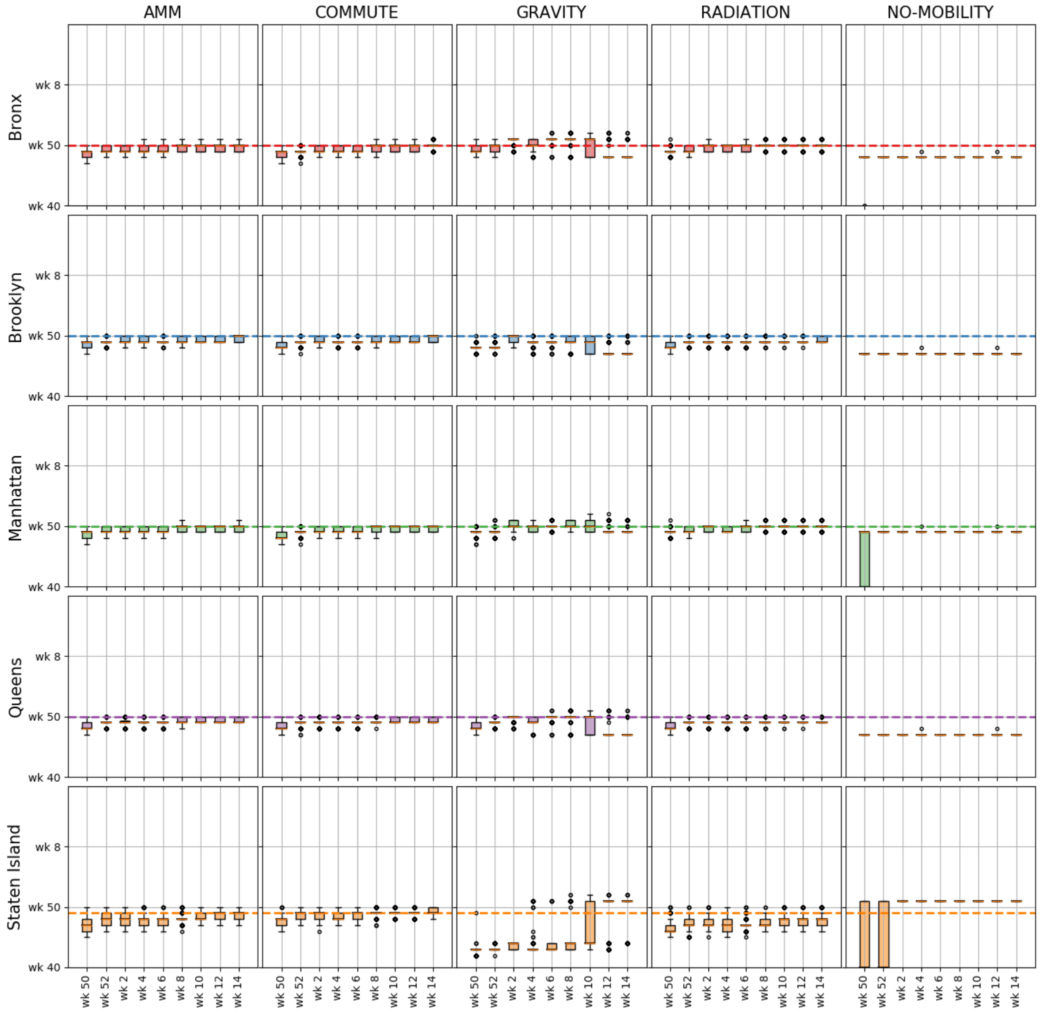

Supplementary Figure 18: The boxplots (median, IQR and whiskers at 1.5IQR) show the posterior prediction of time to onset (we defined onset to be 10% of the peak intensity) for 5 boroughs from 5 models at different prediction horizons (n=1000). The horizontal dashed line represents the ground truth (NYC, 2016-17 season).

Season 2017-2018

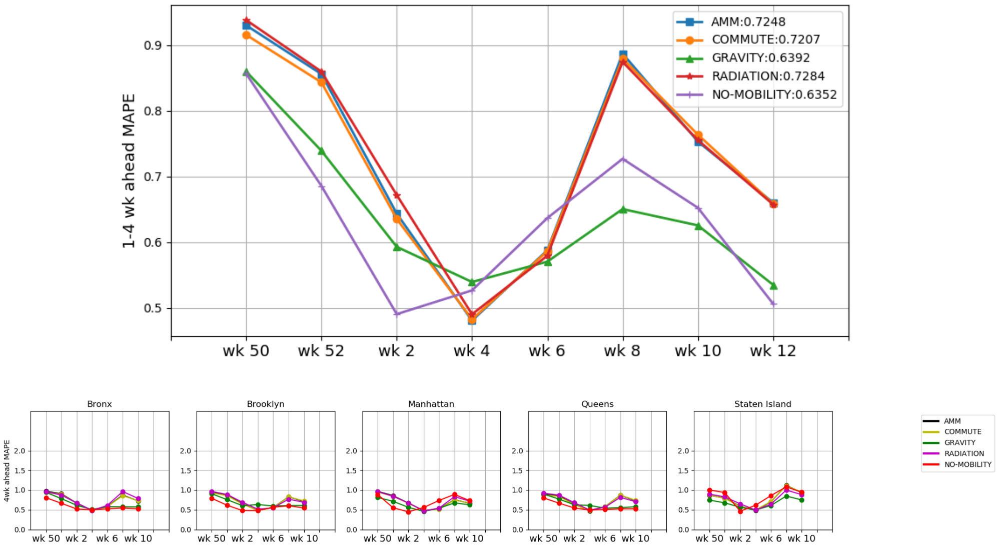

Supplementary Figure 19: Top: Mean absolute prediction error (MAPE) for 4 week look ahead prediction, averaging over 4 weeks and 5 boroughs at prediction horizon from week 50 of 2017 to week 12 of 2018 (NYC). Bottom: MAPE for 4 week look ahead prediction for each borough separately (NYC, 2017-18 season).

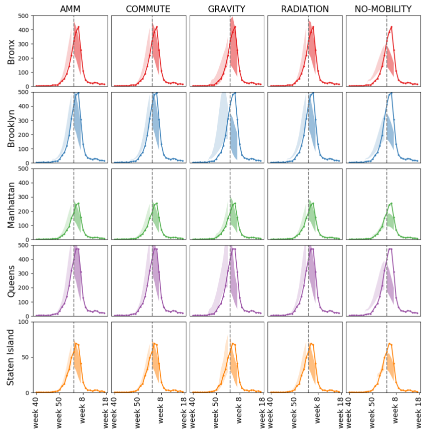

(a) Calibration and prediction at pre-peak

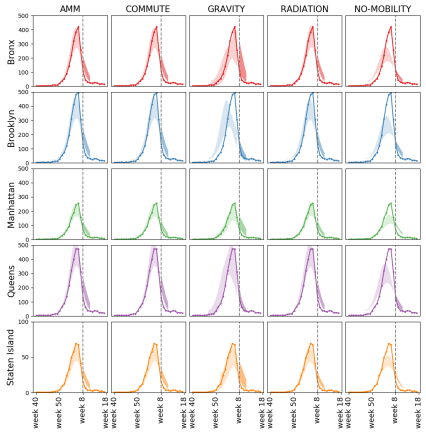

(b) Calibration and prediction at post-peak

Supplementary Figure 20: Pre and post peak 4 week ahead 90% prediction interval for 5 models (NYC, 2017-18 season).

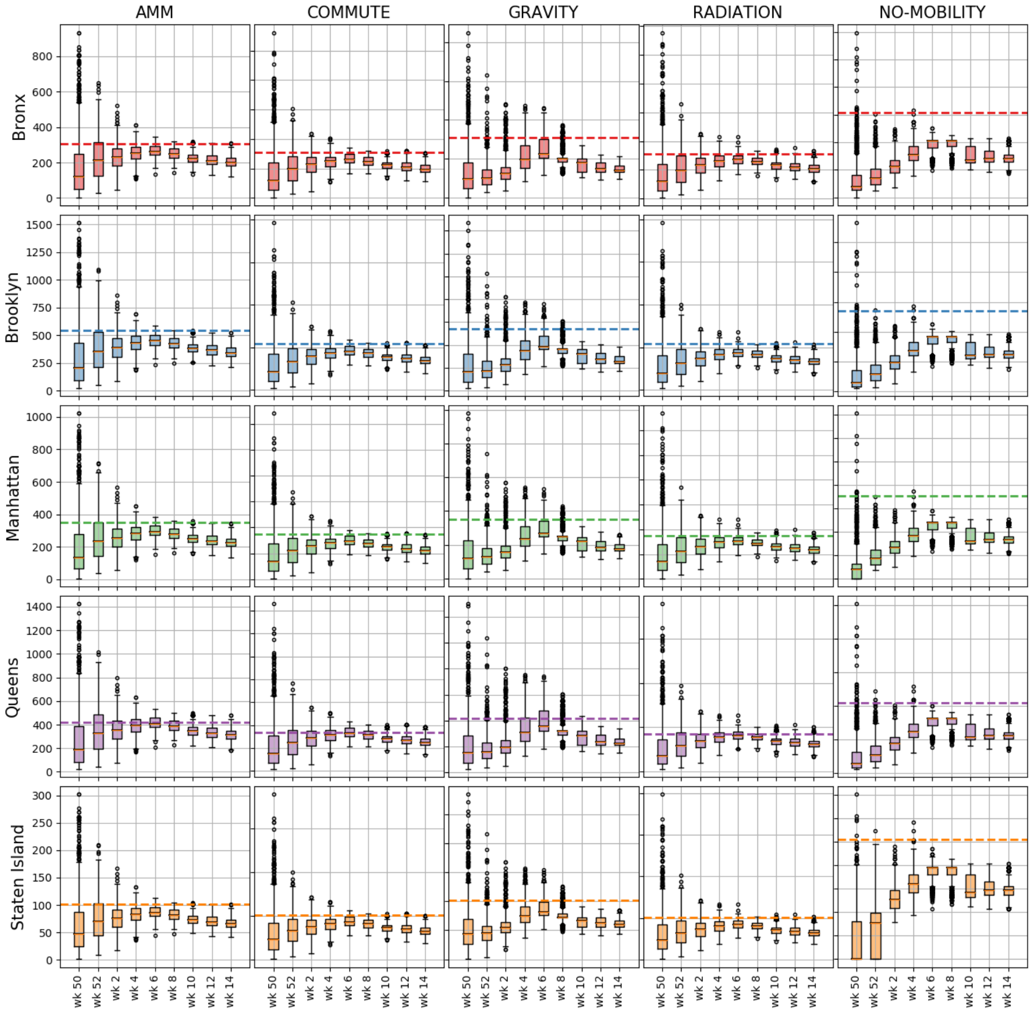

Supplementary Figure 21: The boxplots (median, IQR and whiskers at 1.5IQR) show the posterior prediction of peak intensity for 5 boroughs from 5 models at different prediction horizons (n=1000). The horizontal dashed line represents the ground truth (NYC, 2017-18 season).

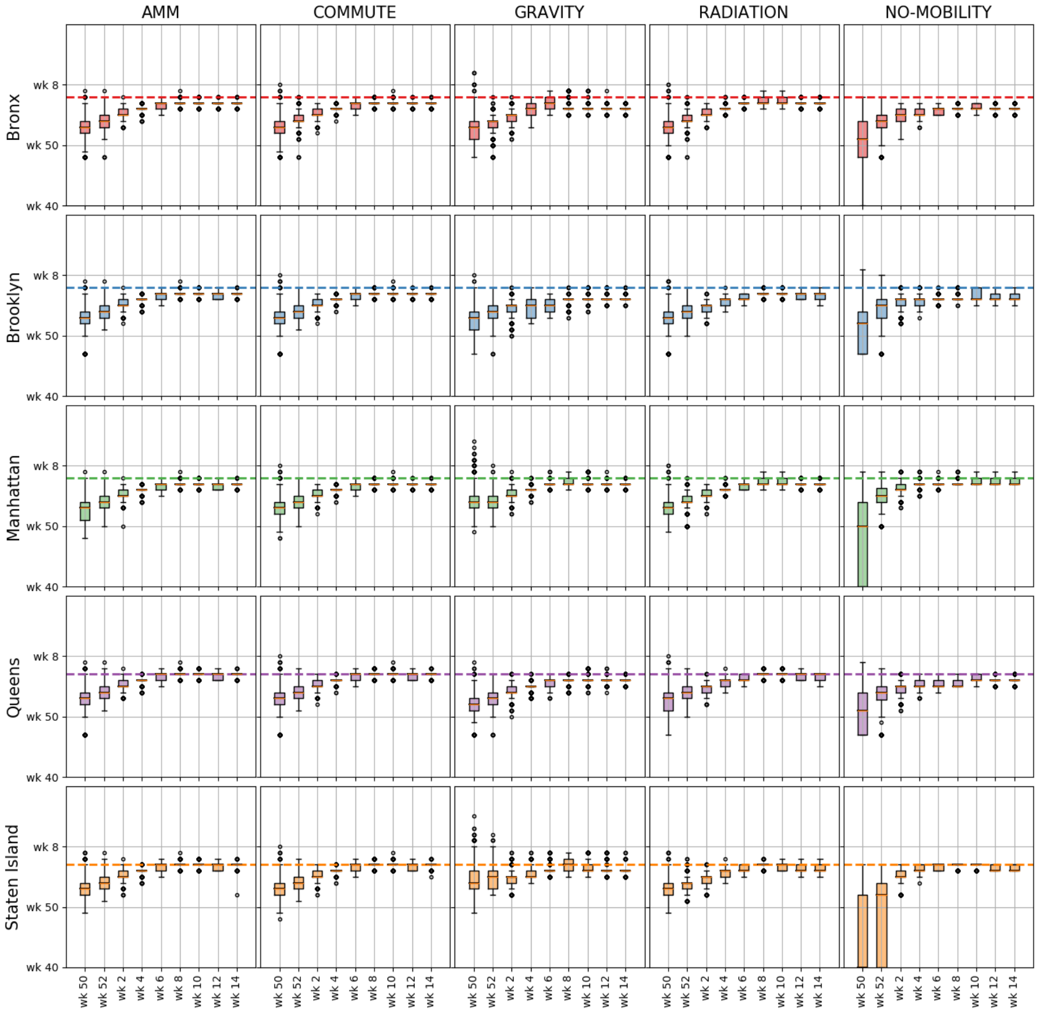

Supplementary Figure 22: The boxplots (median, IQR and whiskers at 1.5IQR) show the posterior prediction of time to peak for 5 boroughs from 5 models at different prediction horizons (n=1000). The horizontal dashed line represents the ground truth (NYC, 2017-18 season).

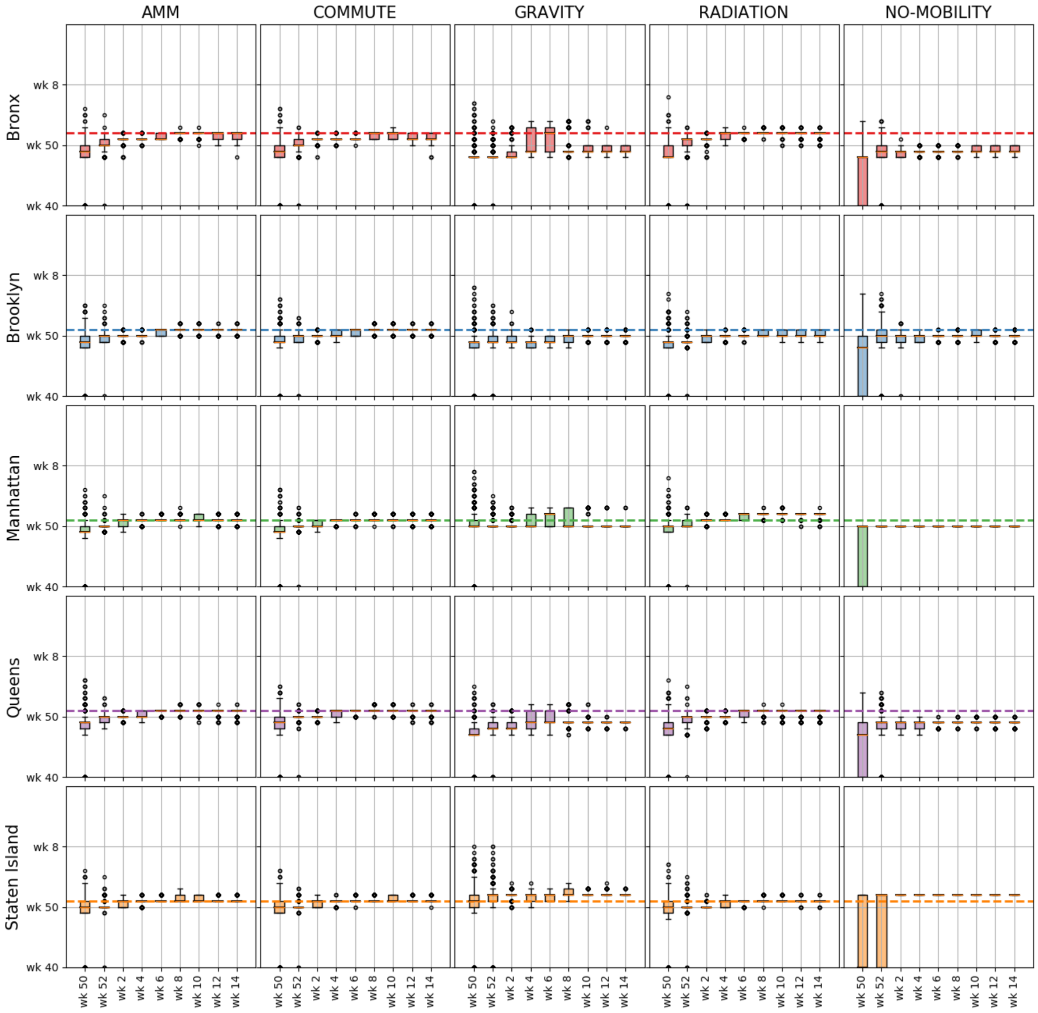

Supplementary Figure 23: The boxplots (median, IQR and whiskers at 1.5IQR) show the posterior prediction of time to onset (we defined onset to be 10% of the peak intensity) for 5 boroughs from 5 models at different prediction horizons (n=1000). The horizontal dashed line represents the ground truth (NYC, 2017-18).

NY-NJ (HHS Region 2), 2016-17

Australia, 2016 season

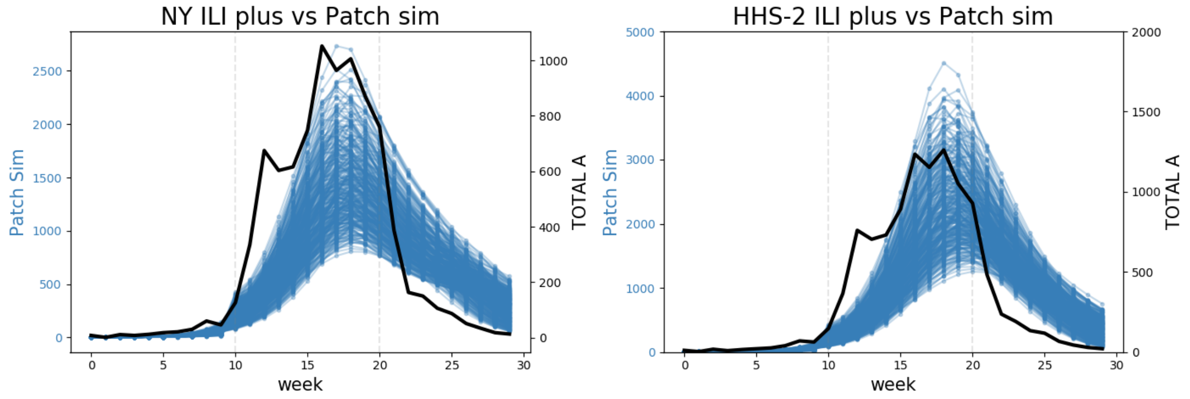

(a) NY state ILIPlus

(b) HHS region 2 ILIPlus

Supplementary Figure 24: The black lines shows the ILI plus ground truth for (left) NY state and (right) HHS 2 region, and the blue curves are the posterior predictions obtained from the calibrated PatchSim model, where the calibration is done only based on the ED visit data from the NYC boroughs (NY, HHS Region 2, 2016-17 season).

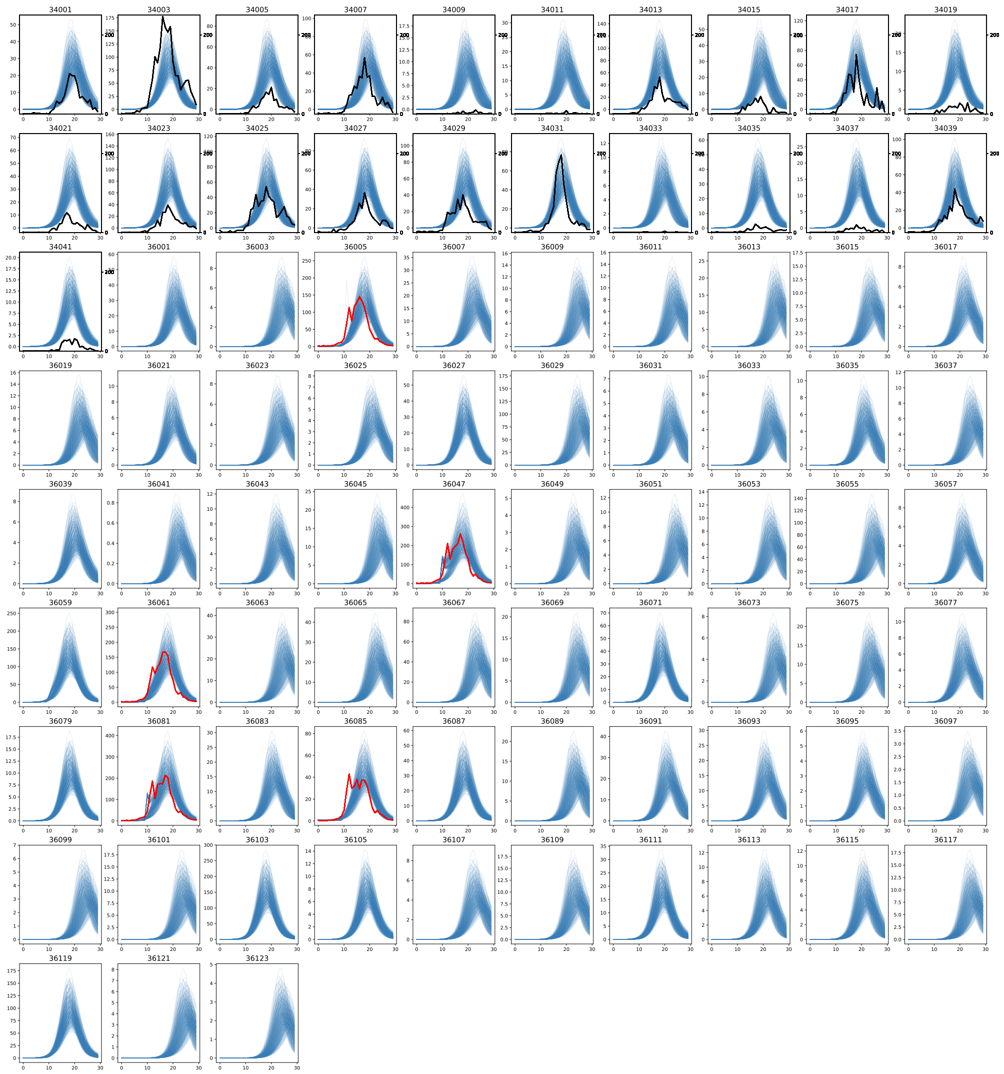

Supplementary Figure 25: Simulation output with observed ground truth for (some of) the counties in New York and New Jersey. The simulation was calibrated only with respect to the NYC counties (NY-NJ, 2016-17 season).

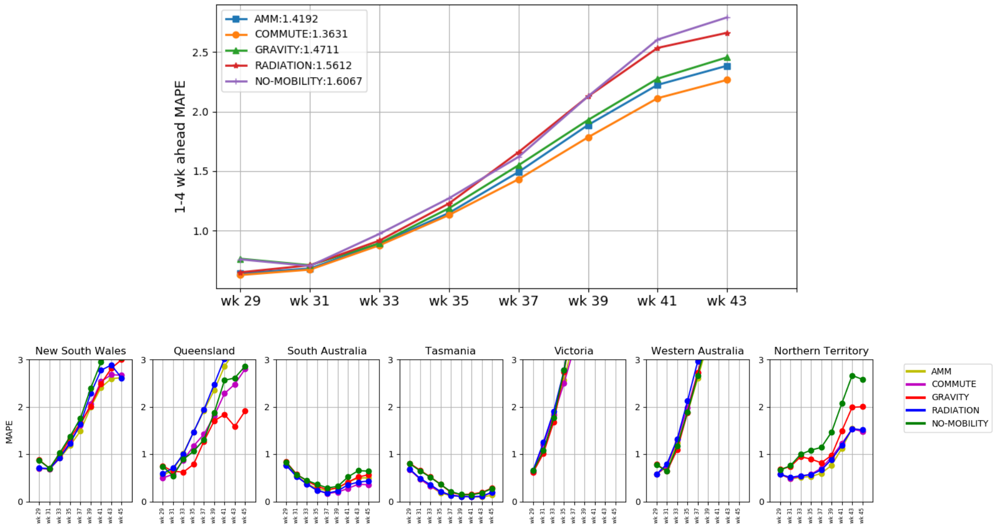

Supplementary Figure 26: Top: Mean absolute prediction error (MAPE) for 4 week look ahead prediction, averaging over 4 weeks and 7 states at prediction horizon from week 29 to week 43 of 2016 for Australia. Bottom: MAPE for 4 week look ahead prediction for each borough separately (Australia, 2016 season).

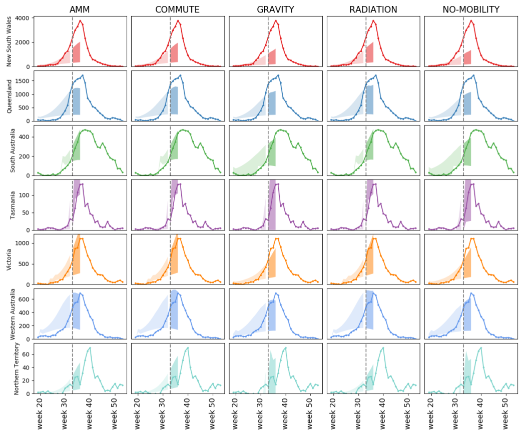

(a) Calibration and prediction at pre-peak

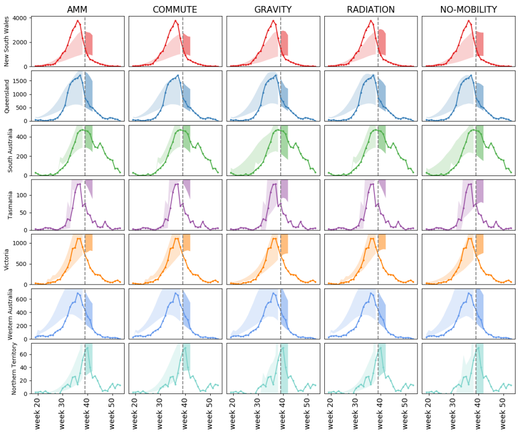

(b) Calibration and prediction at post-peak

Supplementary Figure 27: Pre and post peak 4 week ahead 90% prediction interval for the four models (Australia, 2016 season

.

Supplementary References

[1] Hugo Barbosa, Marc Barthelemy, Gourab Ghoshal, Charlotte R James, Maxime Lenormand, Thomas Louail, Ronaldo Menezes, José J Ramasco, Filippo Simini, and Marcello Tomasini. Human mobility: Models and applications. *Physics Reports*, 2018.

[2] Yves-Alexandre de Montjoye, Zbigniew Smoreda, Romain Trinquart, Cezary Ziemlicki, and Vincent D Blondel. D4d-senegal: the second mobile phone data for development challenge. *arXiv preprint arXiv:1407.4885*, 2014.

[3] Amy Wesolowski, Taimur Qureshi, Maciej F Boni, Pål Roe Sundsøy, Michael A Johansson, Syed Basit Rasheed, Kenth Engø-Monsen, and Caroline O Buckee. Impact of human mobility on the emergence of dengue epidemics in pakistan. *Proceedings of the National Academy of Sciences*, 112(38):11887–11892, 2015.

[4] Amy Wesolowski, Nathan Eagle, Andrew J Tatem, David L Smith, Abdisalan M Noor, Robert W Snow, and Caroline O Buckee. Quantifying the impact of human mobility on malaria. *Science*, 338(6104):267–270, 2012.

[5] Amy Wesolowski, Elisabeth zu Erbach-Schoenberg, Andrew J Tatem, Christopher Lourenço, Cecile Viboud, Vivek Charu, Nathan Eagle, Kenth Engø-Monsen, Taimur Qureshi, Caroline O Buckee, et al. Multinational patterns of seasonal asymmetry in human movement influence infectious disease dynamics. *Nature communications*, 8(1):2069, 2017.

[6] Amy Wesolowski, Caroline O Buckee, Kenth Engø-Monsen, and CJE Metcalf. Connecting mobility to infectious diseases: the promise and limits of mobile phone data. *The Journal of infectious diseases*, 214(suppl_4):S414–S420, 2016.

[7] Stephen Eubank, Hasan Guclu, VS Anil Kumar, Madhav V Marathe, Aravind Srinivasan, Zoltan Toroczkai, and Nan Wang. Modelling disease outbreaks in realistic urban social networks. *Nature*, 429(6988):180, 2004.

[8] Michele Tizzoni, Paolo Bajardi, Adeline Decuyper, Guillaume Kon Kam King, Christian M Schneider, Vincent Blondel, Zbigniew Smoreda, Marta C González, and Vittoria Colizza. On the use of human mobility proxies for modeling epidemics. *PLoS computational biology*, 10(7):e1003716, 2014.

[9] Paolo Bajardi, Chiara Poletto, Jose J Ramasco, Michele Tizzoni, Vittoria Colizza, and Alessandro Vespignani. Human mobility networks, travel restrictions, and the global spread of 2009 h1n1 pandemic. *PloS one*, 6(1):e16591, 2011.

[10] Dirk Brockmann, Vincent David, and Alejandro Morales Gallardo. Human mobility and spatial disease dynamics. *Reviews of nonlinear dynamics and complexity*, 2:1–24, 2009.

[11] Philip Cooley, Shawn Brown, James Cajka, Bernadette Chasteen, Laxminarayana Ganapathi, John Grefenstette, Craig R Hollingsworth, Bruce Y Lee, Burton Levine, William D Wheaton, et al. The role of subway travel in an influenza epidemic: a new york city simulation. *Journal of Urban Health*, 88(5):982, 2011.

[12] Duygu Balcan, Vittoria Colizza, Bruno Gonçalves, Hao Hu, José J Ramasco, and Alessandro Vespignani. Multiscale mobility networks and the spatial spreading of infectious diseases. *Proceedings of the National Academy of Sciences*, pages pnas–0906910106, 2009.

[13] Marco Ajelli, Bruno Gonçalves, Duygu Balcan, Vittoria Colizza, Hao Hu, José J Ramasco, Stefano Merler, and Alessandro Vespignani. Comparing large-scale computational approaches to epidemic modeling: agent-based versus structured metapopulation models. *BMC infectious diseases*, 10(1):190, 2010.

[14] Michele Tizzoni, Kaiyuan Sun, Diego Benusiglio, Márton Karsai, and Nicola Perra. The scaling of human contacts and epidemic processes in metapopulation networks. *Scientific reports*, 5:15111, 2015.

[15] Craig J McGowan, Matthew Biggerstaff, Michael Johansson, Karyn M Apfeldorf, Michal Ben-Nun, Logan Brooks, Matteo Convertino, Madhav Erraguntla, David C Farrow, John Freeze, et al. Collaborative efforts to forecast seasonal influenza in the united states, 2015–2016. *Scientific reports*, 9(1):683, 2019.

[16] Sarah C Kramer and Jeffrey Shaman. Development and validation of influenza forecasting for 64 temperate and tropical countries. *PLoS computational biology*, 15(2):e1006742, 2019.

[17] Nicholas G Reich, Logan C Brooks, Spencer J Fox, Sasikiran Kandula, Craig J McGowan, Evan Moore, Dave Osthus, Evan L Ray, Abhinav Tushar, Teresa K Yamana, et al. A collaborative multiyear, multimodel assessment of seasonal influenza forecasting in the united states. *Proceedings of the National Academy of Sciences*, 116(8):3146–3154, 2019.

[18] Lijing Wang, Jiangzhuo Chen, and Madhav Marathe. Defsi: Deep learning based epidemic forecasting with synthetic information. *Proceedings of the 30th innovative Applications of Artificial Intelligence (IAAI)*, 2019.

[19] Cécile Viboud and Alessandro Vespignani. The future of influenza forecasts. *Proceedings of the National Academy of Sciences*, 116(8):2802–2804, 2019.

[20] Matthew Biggerstaff, David Alper, Mark Dredze, Spencer Fox, Isaac Chun-Hai Fung, Kyle S Hickmann, Bryan Lewis, Roni Rosenfeld, Jeffrey Shaman, Ming-Hsiang Tsou, et al. Results from the centers for disease control and prevention's predict the 2013–2014 influenza season challenge. *BMC infectious diseases*, 16(1):357, 2016.

[21] Jean-Paul Chretien, Dylan George, Jeffrey Shaman, Rohit A Chitale, and F Ellis McKenzie. Influenza forecasting in human populations: a scoping review. *PloS one*, 9(4):e94130, 2014.

[22] Elaine O Nsoesie, John S Brownstein, Naren Ramakrishnan, and Madhav V Marathe. A systematic review of studies on forecasting the dynamics of influenza outbreaks. *Influenza and other respiratory viruses*, 8(3):309–316, 2014.

[23] Shihao Yang, Mauricio Santillana, and Samuel C Kou. Accurate estimation of influenza epidemics using google search data via argo. *Proceedings of the National Academy of Sciences*, 112(47):14473–14478, 2015.

[24] Mauricio Santillana, André T Nguyen, Mark Dredze, Michael J Paul, Elaine O Nsoesie, and John S Brownstein. Combining search, social media, and traditional data sources to improve influenza surveillance. *PLoS computational biology*, 11(10):e1004513, 2015.

[25] Cécile Viboud, Vivek Charu, Donald Olson, Sébastien Ballesteros, Julia Gog, Farid Khan, Bryan Grenfell, and Lone Simonsen. Demonstrating the use of high-volume electronic medical claims data to monitor local and regional influenza activity in the us. *PloS one*, 9(7):e102429, 2014.

[26] Jeffrey Shaman and Alicia Karspeck. Forecasting seasonal outbreaks of influenza. *Proceedings of the National Academy of Sciences*, 109(50):20425–20430, 2012.

[27] Sen Pei, Sasikiran Kandula, Wan Yang, and Jeffrey Shaman. Forecasting the spatial transmission of influenza in the united states. *Proceedings of the National Academy of Sciences*, page 201708856, 2018.

[28] Wan Yang, Donald R Olson, and Jeffrey Shaman. Forecasting influenza outbreaks in boroughs and neighborhoods of new york city. *PLoS computational biology*, 12(11):e1005201, 2016.

[29] Filippo Simini, Marta C González, Amos Maritan, and Albert-László Barabási. A universal model for mobility and migration patterns. *Nature*, 484(7392):96–100, 2012.

[30] Economic development regions of new york state. `https://www.osc.state.ny.us/files/local-government/publications/pdf/economic_development_regions.pdf`. (Accessed on 12/08/2020).
