# Supplementary material for: Forecasting influenza activity using machine-learned mobility map
Source: Nat Commun. 2021 Feb 9;12:726. doi: 10.1038/s41467-021-21018-5 (PMC7873234; doi:10.1038/s41467-021-21018-5)
Supplement: Supplementary file 3 — Reporting Summary [file 41467_2021_21018_MOESM3_ESM.pdf]

nature research

Corresponding author(s): Adam Sadilek, Srinivasan Venkatramanan

Last updated by author(s): Dec 9, 2020

Reporting Summary

Nature Research wishes to improve the reproducibility of the work that we publish. This form provides structure for consistency and transparency in reporting. For further information on Nature Research policies, see our Editorial Policies and the Editorial Policy Checklist.

Statistics

For all statistical analyses, confirm that the following items are present in the figure legend, table legend, main text, or Methods section.

n/a	Confirmed	
☒	☐	The exact sample size (*n*) for each experimental group/condition, given as a discrete number and unit of measurement
☒	☐	A statement on whether measurements were taken from distinct samples or whether the same sample was measured repeatedly
☐	☒	The statistical test(s) used AND whether they are one- or two-sided<br>*Only common tests should be described solely by name; describe more complex techniques in the Methods section.*
☒	☐	A description of all covariates tested
☐	☒	A description of any assumptions or corrections, such as tests of normality and adjustment for multiple comparisons
☐	☒	A full description of the statistical parameters including central tendency (e.g. means) or other basic estimates (e.g. regression coefficient) AND variation (e.g. standard deviation) or associated estimates of uncertainty (e.g. confidence intervals)
☐	☒	For null hypothesis testing, the test statistic (e.g. *F*, *t*, *r*) with confidence intervals, effect sizes, degrees of freedom and *P* value noted<br>*Give P values as exact values whenever suitable.*
☐	☒	For Bayesian analysis, information on the choice of priors and Markov chain Monte Carlo settings
☒	☐	For hierarchical and complex designs, identification of the appropriate level for tests and full reporting of outcomes
☒	☐	Estimates of effect sizes (e.g. Cohen's *d*, Pearson's *r*), indicating how they were calculated

Our web collection on statistics for biologists contains articles on many of the points above.

Software and code

Policy information about availability of computer code

Data collection	The Google Aggregated Mobility Research Dataset was collected, anonymized and aggregated by commercial code belonging to Google LLC.
Data analysis	Disease simulations were conducted using openly available code for simulation engine PatchSim (https://github.com/NSSAC/PatchSim). Custom code were developed for calibration, forecasting and evaluation and are available at https://github.com/NSSAC/AMMFluForecasting. They are provided in Code availability section of the paper with respective DOIs.

For manuscripts utilizing custom algorithms or software that are central to the research but not yet described in published literature, software must be made available to editors and reviewers. We strongly encourage code deposition in a community repository (e.g. GitHub). See the Nature Research guidelines for submitting code & software for further information.

Data

Policy information about availability of data

All manuscripts must include a data availability statement. This statement should provide the following information, where applicable:

- Accession codes, unique identifiers, or web links for publicly available datasets
- A list of figures that have associated raw data
- A description of any restrictions on data availability

Emergency department visits in New York City (NYC) related to Influenza-like Illness were obtained from NYC EpiQuery Syndromic Surveillance portal (https://a816-healthpsi.nyc.gov/epiquery/). Influenza-A positive percentages were obtained from US CDC FluView (https://gis.cdc.gov/grasp/fluview/fluportaldashboard.html). Lab tested flu positive counts for New Jersey were obtained from NJ State Department of Health webpage (https://www.nj.gov/health/cd/statistics/flu-stats). Influenza positive counts for Australia were obtained from Australian Government Department of Health's website for National Notifiable Diseases Surveillance System (http://www9.health.gov.au/cda/source/pub_influ.cfm). Commuter flows for New York and New Jersey were obtained from American Community Survey (https://www.census.gov/data/tables/time-series/demo/commuting/commuting-flows.html). Interstate commuters for Australia were obtained from the Australian

Labour Market Statistics (https://www.abs.gov.au/AUSSTATS/abs@.nsf/Previousproducts/6105.0Feature%20Article1Oct%202008). County population sizes for NY and NJ were obtained from US Census Bureau (https://www.census.gov/topics/population.html). State and territory population sizes for Australia were obtained from Australian Bureau of Statistics (https://www.abs.gov.au/statistics/people/population). Preprocessed versions of the above datasets used in the simulation are provided in the code repository (https://github.com/NSSAC/AMMFluForecasting). The Google Aggregated Mobility Research Dataset used for this study is available with permission from Google LLC.

Field-specific reporting

Please select the one below that is the best fit for your research. If you are not sure, read the appropriate sections before making your selection.

☐ Life sciences ☒ Behavioural & social sciences ☐ Ecological, evolutionary & environmental sciences

For a reference copy of the document with all sections, see nature.com/documents/nr-reporting-summary-flat.pdf

Behavioural & social sciences study design

All studies must disclose on these points even when the disclosure is negative.

Study description	The study aims to measure the utility of various aggregate mobility datasets in forecasting short-term influenza incidence trends at various spatial scales. The study is quantitative in nature and combines disease monitoring collected via public health departments and aggregate mobility flows.
Research sample	The Google Aggregated Mobility Research Dataset contains anonymized mobility flows aggregated over users who have turned on the Location History setting, which is off by default. This is similar to the data used to show how busy certain types of places are in Google Maps — helping identify when a local business tends to be the most crowded. The dataset aggregates flows of people from region to region. To produce this dataset, machine learning is applied to logs data to automatically segment it into semantic trips. To provide strong privacy guarantees, all trips were anonymized and aggregated using a differentially private mechanism to aggregate flows over time (see https://policies.google.com/technologies/anonymization). This research is done on the resulting heavily aggregated and differentially private data. No individual user data was ever manually inspected, only heavily aggregated flows of large populations were handled. We used this datasets restricted to the US states of NY, NJ and Australia, since it represents the level of connectivity within the regions of interest.
Sampling strategy	No explicit sample size calculations were performed. We add a Laplacian noise to the number of unique users for each location pair, for each week. All metrics for which the noisy number of users is lower than 100 are then removed. These are described in the Methods section of the manuscript.
Data collection	Data was collected from mobile phones of users who have turned on the Location History setting, which is off by default. During data collection, the researchers were not present with the participant and were blinded to the experimental conditions and research hypothesis.
Timing	For the results in the main paper, we used weekly aggregated mobility flows from MMWR week 40 of 2016 to MMWR week 39 of 2017 (i.e., 2016-17 influenza season) for the US based study. For Australia the corresponding time period spanned from May to December 2016. Additional results in Supplementary material used flows corresponding to 2015-16 and 2017-18 influenza season in the United States.
Data exclusions	No data were excluded from the study.
Non-participation	Only users who opted in to Location History are aggregated into the population-level flows.
Randomization	Participants were not allocated to experimental groups.

Reporting for specific materials, systems and methods

We require information from authors about some types of materials, experimental systems and methods used in many studies. Here, indicate whether each material, system or method listed is relevant to your study. If you are not sure if a list item applies to your research, read the appropriate section before selecting a response.

Materials & experimental systems

n/a	Involved in the study
☒	☐ Antibodies
☒	☐ Eukaryotic cell lines
☒	☐ Palaeontology and archaeology
☒	☐ Animals and other organisms
☒	☐ Human research participants
☒	☐ Clinical data
☒	☐ Dual use research of concern

Methods

n/a	Involved in the study
☒	☐ ChIP-seq
☒	☐ Flow cytometry
☒	☐ MRI-based neuroimaging
